# Comparative analysis of mycobacterium and related actinomycetes yields insight into the evolution of *mycobacterium tuberculosis *pathogenesis

**DOI:** 10.1186/1471-2164-13-120

**Published:** 2012-03-28

**Authors:** Abigail Manson McGuire, Brian Weiner, Sang Tae Park, Ilan Wapinski, Sahadevan Raman, Gregory Dolganov, Matthew Peterson, Robert Riley, Jeremy Zucker, Thomas Abeel, Jared White, Peter Sisk, Christian Stolte, Mike Koehrsen, Robert T Yamamoto, Milena Iacobelli-Martinez, Matthew J Kidd, Andreia M Maer, Gary K Schoolnik, Aviv Regev, James Galagan

**Affiliations:** 1 Broad Institute, 7 Cambridge Center, Cambridge, MA 02142, USA; 2 DOE Joint Genome Institute, Walnut Creek, CA, USA; 3 Department of Biomedical Engineering, Boston University, Boston, MA, USA; 4 Departments of Microbiology and National Emerging Infectious Diseases Laboratories, Boston University, Boston, MA, USA; 5 VIB Department of Plant Systems Biology, Ghent University, Technologiepark 927, 9052 Ghent, Belgium; 6 Stanford University, Palo Alto, CA, USA; 7 FLIR, Chem-Bio Detection, 505 Coast Boulevard South, Suite 309, La Jolla, CA 92037, USA; 8 Department of Systems Biology, Harvard Medical School, 200 Longwood Ave., Boston, MA 02115, USA; 9 The Broad Institute, 7 Cambridge Center, Cambridge, MA 02142, USA

**Keywords:** Comparative genomics, M. tuberculosis, SYNERGY, Small RNAs, Lipid metabolism, Molybdopterin, DNA repair

## Abstract

**Background:**

The sequence of the pathogen *Mycobacterium tuberculosis *(*Mtb*) strain *H37Rv *has been available for over a decade, but the biology of the pathogen remains poorly understood. Genome sequences from other *Mtb *strains and closely related bacteria present an opportunity to apply the power of comparative genomics to understand the evolution of *Mtb *pathogenesis. We conducted a comparative analysis using 31 genomes from the Tuberculosis Database (TBDB.org), including 8 strains of *Mtb *and *M. bovis*, 11 additional Mycobacteria, 4 Corynebacteria, 2 Streptomyces, *Rhodococcus jostii RHA1, Nocardia farcinia, Acidothermus cellulolyticus, Rhodobacter sphaeroides, Propionibacterium acnes*, and *Bifidobacterium longum*.

**Results:**

Our results highlight the functional importance of lipid metabolism and its regulation, and reveal variation between the evolutionary profiles of genes implicated in saturated and unsaturated fatty acid metabolism. It also suggests that DNA repair and molybdopterin cofactors are important in pathogenic Mycobacteria. By analyzing sequence conservation and gene expression data, we identify nearly 400 conserved noncoding regions. These include 37 predicted promoter regulatory motifs, of which 14 correspond to previously validated motifs, as well as 50 potential noncoding RNAs, of which we experimentally confirm the expression of four.

**Conclusions:**

Our analysis of protein evolution highlights gene families that are associated with the adaptation of environmental Mycobacteria to obligate pathogenesis. These families include fatty acid metabolism, DNA repair, and molybdopterin biosynthesis. Our analysis reinforces recent findings suggesting that small noncoding RNAs are more common in Mycobacteria than previously expected. Our data provide a foundation for understanding the genome and biology of *Mtb *in a comparative context, and are available online and through TBDB.org.

## Background

Tuberculosis is still a major killer worldwide, causing an estimated 2-3 million deaths per year [1]. The sequence of the pathogen *Mycobacterium tuberculosis *(*Mtb*) strain *H37Rv *has been available for a decade [2,3], but the biology of the pathogen remains poorly understood. Available genome sequences from *Mtb *strains and other closely related Mycobacteria present an opportunity to bring the power of comparative genomics to the study of *Mtb*.

**Figure 1:**
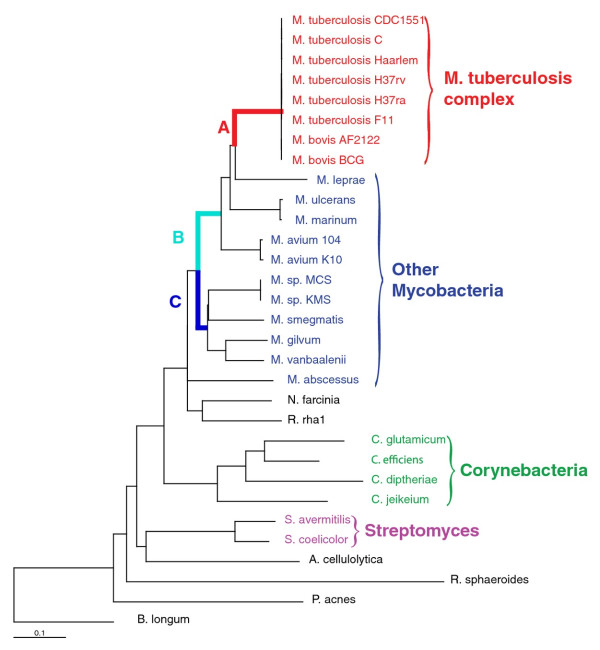
**Phylogenetic Tree based on uniform SYNERGY orthogroups, scaled by phylogenetic distance**. The labels A-C indicate the branches selected for further analysis in our d_N_/d_S _analysis (A: The branch leading to the *Mtb *cluster; B: The branch leading to the pathogenic Mycobacteria; C: The branch leading to the non-pathogenic, soil-dwelling Mycobacteria). See **Methods **for details on the phylogenetic tree construction.

We report here the results of a comparative analysis of 31 publicly available genomes (http://www.tbdb.org, Figure 1, Table 1). These include eight closely related members of the *Mtb *complex that can cause tuberculosis disease, (two *M. bovis *strains and six *Mtb *strains). Another 11 additional Mycobacteria range from obligate parasites to free-living soil bacteria: *M. leprae *and *M. avium subsp. Paratuberculosis K10*, the causative agents of leprosy and paratuberculosis respectively, are pathogenic and require hosts to survive; *M. ulcerans, M. marinum, M. avium 104*, and *M. abscessus *have the potential to be pathogenic but can survive outside the confines of a host; *M. vanbaalenii, M. sp. KMS, M. sp. MCS*, and *M. gilvum *are free-living soil bacteria which are known to degrade a variety of compounds including polycyclic aromatic hydrocarbons (PAH), and are useful in bioremediation efforts. Thus, the Mycobacteria included in our dataset span an ecological range from host-dependent pathogens to soil bacteria, allowing us to study multiple evolutionary transitions.

**Table 1:** Summary of Organisms

Organism	Patho-genic	Host required	Description	Reference
*Mycobacterium tuberculosis H37Rv*	Y	Y	Causes TB; Laboratory strain	[2]

*Mycobacterium tuberculosis H37Ra*	Y	Y	Causes TB; Avirulent sister strain to *H37Rv*	[4]

*Mycobacterium tuberculosis F11 (ExPEC)*	Y	Y	Causes TB; isolated from TB patient in S. Africa	[5]

*Mycobacterium bovis BCG str. Pasteur 1173P2*	Y	Y	Causes bovine TB; attenuated vaccine strain	[6]

*Mycobacterium bovis AF2122/97*	Y	Y	Causes bovine TB	[7]

*Mycobacterium tuberculosis Haarlem*	Y	Y	Causes TB; MDR strain	[5]

*Mycobacterium tuberculosis C*	Y	Y	Causes TB; isolated in NY City	[5]

*Mycobacterium tuberculosis CDC1551*	Y	Y	Causes TB; highly contagious & virulent strain	[8]

*Mycobacterium ulcerans AGY99*	Y		Causes Buruli ulcer	[9]

*Mycobacterium marinum*	Y		From fish; Skin lesions in human	[10]

*Mycobacterium leprae TN*	Y	Y	Causes leprosy	[11]

*Mycobacterium avium 104*	Y		Opportunistic pathogen; can causeTB-type pulmonary infection	[12]

*Mycobacterium avium subsp. Paratuberculosis K-10*	Y	Y	Causes paratuberculosis; obligate pathogen of cattle	[13]

*Mycobacterium sp. MCS*			Soil bacteria; degrades PAH	[14]

*Mycobacterium sp. KMS*			Soil bacteria; degrades PAH	[14]

*Mycobacterium smegmatis MC2155*	Y		Widely used model for *Mtb *isolated from human smegma; causes soft tissue lesions	[12]

*Mycobacterium vanbaalenii PYR-1*			Soil bacteria; degrades PAH	[14]

*Mycobacterium gilvum*			Soil bacteria; Degrades PAH + wide variety of organic compounds	[14]

*Mycobacterium abscessus*	Y		Skin & soft tissue infections	[15]

*Rhodococcus jostii RHA1*			Soil bacteria important for biofuels research and bioremediation; degrades PCB + wide variety of organic compounds	[16]

*Nocardia farcinica IFM 10152*	Y		Causes nocardiosis	[17]

*Corynebacterium glutamicum ATCC 13032*			Produces amino acids (Glu)	[18]

*Corynebacterium efficiens YS-314*			Produces amino acids (Glu)	[19]

*Corynebacterium diphtheriae NCTC13129*	Y		Causes diphtheria	[20]

*Corynebacterium jeikeium K411*	Y		Causes nocosomial infections	[21]

*Streptomyces avermitilis MA-4680*			Soil bacteria;antibiotic-producing	[22]

*Streptomyces coelicolor A3(2)*			Soil bacteria;antibiotic producing	[23]

*Acidothermus cellulolyticus 11B*			Hot springs of Yellowstone	[24]

*Rhodobacter sphaeroides*			Gram -, motile; photosyn.; fixes N_2_	[14]

*Propionibacterium Acnes KPA171202*	Y		Causes acne	[25]

*Bifidobacterium Longum NCC2705*			Digestive track commensal; yogurt	[26]

To gain further insight into the Mycobacterium cluster, we also included a related *Rhodococcus *(also involved in bioremediation), a pathogenic *Nocardia*, four *Corynebacteria *(two pathogens and two that are commercially useful in amino acid production), two *Streptomyces *(antibiotic-producing soil bacteria), *Acidothermus cellulolyticus *(a thermophilic actinobacteria from the hot springs of Yellowstone), *Propionibacterium acnes *(causative agent of common acne), and *Bifidobacterium longum *(a digestive track commensal often found in yogurt). We extend this comparative analysis to other more distantly related *Actinobacteria *to yield additional insight into evolutionary trends.

We examined protein evolution across these 31 organisms, both at the nucleotide level and at the level of protein families, including studying gene families associated with the transition from nonpathogenic soil-dwelling bacteria to obligate pathogens. Our results highlight the importance of lipid metabolism and its regulation, and reveal differences in the evolutionary profiles for genes implicated in saturated and unsaturated fatty acid metabolism. Our analysis also suggests that DNA repair and molybdopterin cofactors are expanded in pathogenic Mycobacteria and *Mtb*.

We also identified highly conserved elements within noncoding regions using whole-genome multiple alignments and gene expression data. These conserved elements include 37 predicted conserved promoter regulatory motifs, of which 14 correspond to previously reported motifs. They also include approximately 50 predicted novel noncoding RNAs. Guided by our computational analysis, we tested and experimentally confirmed the expression of 4 novel small RNAs in *Mtb*.

The results of our analyses are available on our website, and provide a foundation for understanding the genome and biology of *Mtb *in a comparative context.

## Results and discussion

### An orthogroup catalogue for Mycobacteria

**Figure 2:**
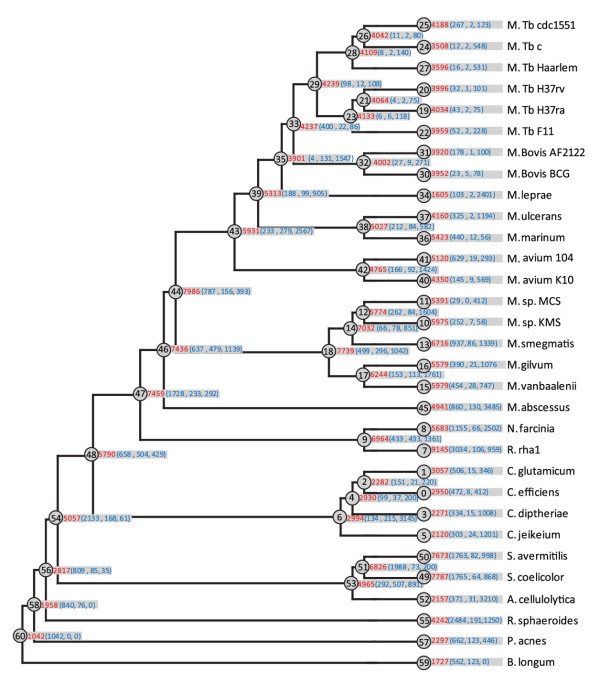
**Summary of SYNERGY results: Number of gains, losses, and duplication at each node**. For each node, the node number is marked in black; the total number of genes present at each node is indicated in red, and the numbers of gains, losses, and duplications are indicated in parenthesis in blue http://www.broadinstitute.org/ftp/pub/seq/msc/pub/SYNERGY/index.html.

We used SYNERGY [27,28] to reconstruct the phylogeny of proteins across all 31 organisms, define sets of orthologs ("orthogroups"), and construct a phylogenetic tree of the genomes (Figure 1). An orthogroup is defined as the set of genes descended from a single common ancestral gene in the last common ancestor of the species under consideration [28], containing both orthologs and possibly paralogs (**Methods**). At each node in the phylogenetic tree, we tabulated orthogroup appearances, duplications, and losses (Figure 2). Figure 2 gives an overview of the evolution of gene families within these species. Full listings of the events tabulated in Figure 2, as well as additional information about each orthogroup, can be found on the Supplementary Information website:

### Tracing the evolution of biological processes

**Figure 3:**
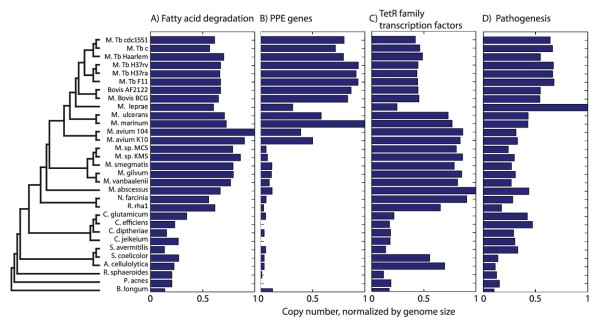
**Evolution of gene categories**. This figure shows several examples of the evolution of metabolic pathways, PFAM domains, and GO term descriptors. Graphics similar to these can be found for each category in the supplementary information website at http://www.broadinstitute.org/ftp/pub/seq/msc/pub/SYNERGY/index.html. a) Fatty acid degradation metabolic pathway. b) PFAM group PF00823 (PPE genes). c) PFAM group PF00440 (tetR family transcription factors). d) GO term 0009405 (pathogenesis.)

To examine the evolution of entire pathways and gene families, we categorized orthogroups according to GO (Gene Ontology) and GO Slim terms [29], PFAM domains [30], metabolic pathways, predicted regulons (sets of genes predicted to be regulated by a common regulatory protein), and groups of genes upregulated under certain lipids (**Methods**). We also looked for orthogroups undergoing positive selection by calculating the ratio of nonsynonymous to synonymous mutations (the d_N_/d_S _ratio). Figure 3 shows several examples of pathway or gene family profiles and the predicted evolutionary events associated with the gene family. The sort of graphic presented in Figure 3 is browsable for every pathway, PFAM, and GO term in our Supplementary Information website. Tables 2 and 3 show the PFAM and GO categories most expanded (with the most orthogroup members) in the *Mtb *clade relative to the non-pathogenic Mycobacteria, and Tables 4 and 5 show those most expanded in the Mycobacteria relative to the non-Mycobacteria.

**Table 2:** 50 PFAM categories most expanded in the *Mtb *clade relative to the non-pathogenic, soil-dwelling Mycobacteria

PFAM name	PFAM ID	p-value^a^	inter-to-intra- centroid difference
^b ^PIN domain	PF01850	4.20E-09	1.10E+01

GHMP kinases C terminal	PF08544	1.00E-08	8.80E+00

DHHA1 domain	PF02272	2.10E-08	6.80E+00

KGG Stress-induced bacterial acidophilic repeat motif	PF10685	6.70E-08	6.20E+00

^b ^Protein of unknown function (DUF1396) *(lipoproteins within cell wall*)	PF07161	4.10E-07	9.20E+00

^b ^*Rv0623*-like transcription factor *(toxin-antitoxin-related)*	PF07704	9.30E-07	1.00E+01

Tetratricopeptide repeat	PF07720	9.90E-07	8.60E+00

PA domain	PF02225	1.00E-06	6.40E+00

^c ^Patatin-like phospholipase	PF01734	1.20E-06	9.20E+00

^e ^Protein of unknown function (DUF1490)	PF07371	1.20E-06	1.00E+01

4 FAD binding domain	PF01565	1.30E-06	6.40E+00

Fumarate reductase/succinate dehydrog. flavoprotein C-term domain	PF02910	1.50E-06	5.90E+00

FIST C domain	PF10442	2.00E-06	1.20E+01

Corticotropin ACTH domain	PF00976	2.30E-06	6.00E+00

^c ^Beta-ketoacyl synthase, N-terminal domain	PF00109	2.40E-06	5.00E+00

IlvB leader peptide	PF08049	4.20E-06	3.50E+00

^c ^Beta-ketoacyl synthase, C-terminal domain	PF02801	4.50E-06	4.80E+00

^c ^Acyl transferase domain	PF00698	4.60E-06	5.20E+00

^b ^PPE family *(antigenic variability)*	PF00823	5.60E-06	1.00E+01

^b ^Proteins of 100 residues with WXG *(esx-related)*	PF06013	7.10E-06	7.90E+00

^b ^Phd YefM *(toxin-antitoxin-related)*	PF02604	7.70E-06	9.00E+00

Ponericin	PF07442	7.70E-06	8.70E+00

^b ^Plasmid stabilization system protein *(toxin-antitoxin-related)*	PF05016	9.90E-06	9.30E+00

Threonine leader peptide	PF08254	1.10E-05	8.90E+00

Toxin 33 Waglerin family	PF08121	2.20E-05	3.20E+00

Phosphatidylethanolamine-binding protein	PF01161	2.20E-05	7.30E+00

^b ^PemK-like protein *(toxin-antitoxin-related)*	PF02452	3.10E-05	9.00E+00

Erythronolide synthase docking	PF08990	5.60E-05	7.30E+00

^d ^Radical SAM superfamily	PF04055	5.80E-05	3.90E+00

^d ^ThiS family	PF02597	6.60E-05	5.60E+00

^b ^Pentapeptide repeats (8 copies)	PF01469	1.20E-04	1.00E+01

Rubredoxin	PF00301	1.30E-04	3.80E+00

^d ^Pterin 4 alpha carbinolamine dehydratase	PF01329	1.50E-04	8.80E+00

Leucine rich repeat N-terminal domain	PF01462	1.60E-04	1.00E+01

^e ^Domain of unknown function (DUF1610)	PF07754	2.10E-04	2.40E+00

SEC-C motif	PF02810	2.30E-04	3.00E+00

^d ^MoaC family	PF01967	2.60E-04	7.10E+00

Berberine and berberine like	PF08031	2.70E-04	9.30E+00

Cytochrome B6-F complex subunit VI (PetL)	PF05115	2.80E-04	9.40E+00

Region found in RelA/SpoT proteins	PF04607	3.00E-04	5.00E+00

Quinolinate phosphoribosyl transferase, C-terminal domain	PF01729	3.30E-04	4.20E+00

Fumarate reductase subunit C	PF02300	3.70E-04	1.00E+01

LHC Antenna complex alpha/beta subunit	PF00556	5.00E-04	3.20E+00

RNPHF zinc finger	PF08080	5.10E-04	6.50E+00

Protein of unknown function (DUF1416)	PF07210	5.20E-04	9.60E+00

PsbJ	PF01788	5.40E-04	3.70E+00

Bacterial transferase hexapeptide (three repeats)	PF00132	5.50E-04	3.00E+00

N Chalcone and stilbene synthases, N-terminal domain	PF00195	6.60E-04	8.40E+00

^d ^MoaE protein	PF02391	8.00E-04	7.80E+00

^b ^Protein of unknown function (DUF1066) *(esx)*	PF06359	8.30E-04	1.10E+01

^a ^Bonferroni-corrected p-value calculated from T-test

^b ^pathogenicity or survival within the host

^c ^Lipid metabolism

^d ^Pterin cofactor biosynthesis

^e ^unknown function

**Table 3:** The 50 GO terms most expanded in the *Mtb *clade relative to the non-pathogenic, soil-dwelling Mycobacteria

GO descriptor	GO term ID	p-value^a^	inter-to-intra centroid difference
4-hydroxy-3-methylbut-2-en-1-yl diphosphate red. activity	GO_0051745	4.80E-08	7.20E+00

dTMP biosynthetic process	GO_0006231	7.30E-07	4.40E+00

response to cAMP	GO_0051591	1.40E-06	1.20E+01

succinate dehydrogenase (ubiquinone) activity	GO_0008177	1.70E-06	5.10E+00

iron ion transport	GO_0006826	2.70E-06	4.70E+00

magnesium ion binding	GO_0000287	2.80E-06	1.70E+00

^c ^fatty-acyl-CoA synthase activity	GO_0004321	5.10E-06	6.50E+00

^c ^acyltransferase activity	GO_0008415	7.60E-06	3.60E+00

transferase activity, transferring alkyl or aryl (other than methyl) groups	GO_0016765	1.30E-05	2.60E+00

^c ^tricarboxylic acid cycle	GO_0006099	1.30E-05	3.40E+00

^d ^Mo-molybdopterin cofactor biosynthetic process	GO_0006777	1.40E-05	6.30E+00

integral to membrane	GO_0016021	2.00E-05	2.00E+00

acid phosphatase activity	GO_0003993	2.50E-05	3.20E+00

phosphatase activity	GO_0016791	3.20E-05	4.10E+00

erythronolide synthase activity	GO_0047879	4.20E-05	8.20E+00

^d ^4-alpha-hydroxytetrahydrobiopterin dehydratase activity	GO_0008124	6.80E-05	6.20E+00

^c ^lipid metabolic process	GO_0006629	6.80E-05	4.70E+00

bacteriochlorophyll biosynthetic process	GO_0030494	7.00E-05	1.30E+00

plasma membrane	GO_0005886	7.10E-05	2.70E+00

^d ^tetrahydrobiopterin biosynthetic process	GO_0006729	1.10E-04	8.80E+00

^c ^lipid biosynthetic process	GO_0008610	1.20E-04	3.50E+00

phosphatidylcholine metabolic process	GO_0046470	1.60E-04	9.50E+00

^c ^geranyltranstransferase activity	GO_0004337	1.60E-04	6.80E+00

cytoplasm	GO_0005737	1.90E-04	1.40E+00

protein transport	GO_0015031	1.90E-04	1.70E+00

guanosine tetraphosphate metabolic process	GO_0015969	2.20E-04	5.00E+00

glyoxylate cycle	GO_0006097	2.20E-04	4.30E+00

phosphoglycolate phosphatase activity	GO_0008967	2.80E-04	4.30E+00

terpenoid biosynthetic process	GO_0016114	3.90E-04	2.80E+00

sulfur metabolic process	GO_0006790	4.10E-04	5.30E+00

4 iron, 4 sulfur cluster binding	GO_0051539	5.00E-04	2.90E+00

succinate dehydrogenase activity	GO_0000104	5.70E-04	4.60E+00

^b ^mycocerosate synthase activity	GO_0050111	5.80E-04	4.10E+00

^c ^phospholipid biosynthetic process	GO_0008654	6.10E-04	2.30E+00

nucleoside metabolic process	GO_0009116	6.30E-04	3.60E+00

^c ^phosphopantetheine binding	GO_0031177	8.20E-04	3.00E+00

adenylate cyclase activity	GO_0004016	8.30E-04	5.50E+00

D-arabinono-1,4-lactone oxidase activity	GO_0003885	9.70E-04	8.40E+00

anaerobic respiration	GO_0009061	9.90E-04	1.10E+01

nodulation	GO_0009877	1.10E-03	7.10E+00

^c ^prenyltransferase activity	GO_0004659	1.10E-03	4.20E+00

^c ^lysophospholipase activity	GO_0004622	1.30E-03	8.50E+00

^c ^acetyl-CoA carboxylase activity	GO_0003989	1.30E-03	2.40E+00

histidinol-phosphatase activity	GO_0004401	2.10E-03	6.50E+00

pyridine nucleotide biosynthetic process	GO_0019363	2.30E-03	5.00E+00

NAD biosynthetic process	GO_0009435	3.30E-03	1.30E+00

lactate fermentation to propionate and acetate	GO_0019652	3.40E-03	3.40E+00

alkylglycerone-phosphate synthase activity	GO_0008609	3.40E-03	7.10E+00

^b ^cyclopropane-fatty-acyl-phospholipid synthase activity	GO_0008825	4.00E-03	5.90E+00

methylcrotonoyl-CoA carboxylase activity	GO_0004485	4.40E-03	3.00E+00

^a^Bonferroni-corrected p-value calculated from T-test

^b ^pathogenicity or survival within the host

^c ^Lipid metabolism

^d ^Cofactor biosynthesis

^e ^unknown function

**Table 4:** The 50 PFAM categories most expanded in the Mycobacteria relative to the non- Mycobacteria

PFAM descriptor	PFAM ID	p-value^a^	inter-to-intra centroid difference
^e ^Protein of unknown function (DUF2599)	PF10783	1.50E-10	8.80E+00

^c ^Cutinase	PF01083	1.60E-10	1.50E+01

^e ^Uncharacterized protein conserved in bacteria (DUF2236)	PF09995	2.20E-10	1.60E+01

^c ^Lpp-LpqN Probable lipoprotein LpqN	PF10738	4.70E-10	1.30E+01

^e ^Domain of unknown function (DUF385)	PF04075	5.70E-10	1.30E+01

^b ^Domain of unk function DUF140 *(yrbE genes in mce operons)*	PF02405	1.60E-09	1.40E+01

Retinal pigment epithelial membrane protein	PF03055	8.50E-09	1.40E+01

^e ^Domain of unknown function (DUF427)	PF04248	1.40E-08	1.60E+01

ABC transporter transmembrane region 2 PF06472	PF06472	1.80E-08	1.10E+01

^b ^Peroxidase *(katG-isoniazid resistance)*	PF00141	4.10E-08	1.20E+01

^b ^mce related protein	PF02470	1.30E-07	1.30E+01

N O-methyltransferase N-terminus	PF02409	1.70E-07	1.70E+01

Activator of Hsp90 ATPase homolog 1-like protein	PF08327	2.40E-07	1.10E+01

Coronavirus nonstructural protein NS1	PF06145	3.00E-07	1.20E+01

^e ^Predicted integral membrane protein (DUF2189)	PF09955	3.10E-07	1.40E+01

^e ^Uncharacterized protein family (UPF0089)	PF03007	3.80E-07	1.50E+01

^b ^Acetyltransf 2 N-acetyltransferase *(inactivates isoniazid)*	PF00797	3.90E-07	1.70E+01

^e ^Domain of unknown function (DUF1957)	PF09210	5.50E-07	7.90E+00

KRAB box	PF01352	7.00E-07	1.70E+01

Prokaryotic acetaldehyde dehydrogenase, dimerisation	PF09290	8.10E-07	9.00E+00

DmpG-like communication domain	PF07836	9.50E-07	1.10E+01

Nuclear transport factor 2 (NTF2) domain	PF02136	1.00E-06	1.30E+01

Wyosine base formation	PF08608	1.10E-06	1.40E+01

AIG2-like family	PF06094	1.30E-06	1.80E+01

^e ^Protein of unknown function (DUF867)	PF05908	1.30E-06	1.50E+01

Phage-related minor tail protein	PF10145	2.10E-06	1.20E+01

^c ^Fatty acid desaturase	PF03405	2.30E-06	1.00E+01

PaaX-like protein	PF07848	2.80E-06	1.50E+01

Adenylate and Guanylate cyclase catalytic domain	PF00211	3.70E-06	1.10E+01

Fibronectin-attachment protein (FAP)	PF07174	3.80E-06	1.30E+01

Leucine Rich Repeat	PF07723	5.50E-06	1.10E+01

2-nitropropane dioxygenase	PF03060	5.70E-06	1.40E+01

^c ^Fatty acid desaturase	PF00487	7.90E-06	1.30E+01

^e ^Protein of unknown function (DUF732)	PF05305	9.10E-06	1.50E+01

^c ^Enoyl-CoA hydratase/isomerase family	PF00378	9.70E-06	1.10E+01

arg-2/CPA1 leader peptide	PF08252	1.00E-05	1.40E+01

^c ^alpha/beta hydrolase fold *(lipases)*	PF07859	1.10E-05	1.50E+01

Cytochrome P450	PF00067	1.40E-05	1.20E+01

^c ^Cyclopropane-fatty-acyl-phospholipid synthase PF02353	PF02353	1.60E-05	1.20E+01

Isoprenylcysteine carboxyl methyltransferase (ICMT) family	PF04140	1.90E-05	1.50E+01

Hydratase/decarboxylase	PF01689	2.50E-05	6.60E+00

PsbJ	PF01788	3.00E-05	9.40E+00

Linocin M18 bacteriocin protein	PF04454	3.40E-05	1.60E+01

Extensin-like protein repeat	PF02095	4.00E-05	1.50E+01

5HT transporter Serotonin (5-HT) neurotransmitter transporter, N-terminus	PF03491	4.00E-05	9.50E+00

^e ^Protein of unknown function (DUF571)	PF04600	4.20E-05	7.70E+00

Tryptophyllin-3 skin active peptide	PF08248	4.90E-05	1.50E+01

AMP-binding enzyme	PF00501	8.10E-05	9.20E+00

^e ^Bacterial protein of unknown function (DUF853)	PF05872	9.90E-05	1.30E+01

^c ^Acyl-ACP thioesterase	PF01643	1.10E-04	1.10E+01

^a^Bonferroni-corrected p-value calculated from T-test

^b ^pathogenicity or survival within the host

^c ^Lipid metabolism

^d ^Cofactor biosynthesis

^e ^unknown function

**Table 5:** The 50 GO terms most expanded in the Mycobacteria relative to the non- Mycobacteria

GO term descriptor	GO term ID	p-value^a^	inter-to-intra-centroid difference
^c ^sterol biosynthetic process	GO:0016126	1.00E-10	1.80E+01

^b ^regulation of apoptosis	GO:0042981	1.10E-10	1.90E+01

^c ^3alpha,7alpha,12alpha-trihydroxy-5beta- cholest-24-enoyl-CoA hydratase activity	GO:0033989	1.40E-10	1.70E+01

^c ^linalool 8-monooxygenase activity	GO:0050056	1.50E-10	1.70E+01

^c ^sterol 14-demethylase activity	GO:0008398	5.10E-09	1.60E+01

^c ^cutinase activity	GO:0050525	5.80E-09	1.60E+01

oxidoreductase activity, acting on NADH or NADPH, nitrogenous group as acceptor	GO:0016657	1.80E-08	1.40E+01

^c ^diacylglycerol O-acyltransferase activity	GO:0004144	2.10E-08	1.70E+01

ligase activity, forming carbon-carbon bonds	GO:0016885	6.10E-08	1.40E+01

indolylacetylinositol arabinosyltransferase activity	GO:0050409	7.90E-08	1.20E+01

^c ^4-hydroxy-2-oxovalerate aldolase activity	GO:0008701	1.00E-07	1.10E+01

arylamine N-acetyltransferase activity	GO:0004060	2.80E-07	1.70E+01

^c ^lipid transport	GO:0006869	5.00E-07	1.70E+01

^c ^lipid biosynthetic process	GO:0008610	5.00E-07	8.50E+00

biphenyl-2,3-diol 1,2-dioxygenase activity	GO:0018583	1.40E-06	9.50E+00

cis-stilbene-oxide hydrolase activity	GO:0033961	1.50E-06	1.20E+01

^c ^acyl-[acyl-carrier-protein] desaturase activity	GO:0045300	1.70E-06	1.00E+01

5-carboxymethyl-2-hydroxymuconic-semialdehyde dehydrog activity	GO:0018480	2.00E-06	1.20E+01

^c ^fatty-acyl-CoA synthase activity	GO:0004321	2.10E-06	1.20E+01

^c ^steroid biosynthetic process	GO:0006694	2.30E-06	1.20E+01

^c ^propanoyl-CoA C-acyltransferase activity	GO:0033814	2.50E-06	1.20E+01

extracellular matrix binding	GO:0050840	2.70E-06	1.30E+01

lipid glycosylation	GO:0030259	6.70E-06	5.60E+00

^d ^coenzyme F420-dependent N5, N10-methenyltetrahydromethanopterinreductase activity	GO:0018537	6.90E-06	1.20E+01

C-terminal protein amino acid methylation	GO:0006481	1.10E-05	1.10E+01

metabolic process	GO:0008152	1.30E-05	4.60E+00

4-oxalocrotonate decarboxylase activity	GO:0047437	1.70E-05	1.10E+01

oxidoreductase activity	GO:0016491	1.80E-05	5.20E+00

oxidation reduction	GO:0055114	1.90E-05	4.80E+00

defense response to bacterium	GO:0042742	2.40E-05	1.60E+01

^b ^cyclopropane-fatty-acyl-phospholipid synthase activity	GO:0008825	2.90E-05	9.80E+00

^c ^3-hydroxy-2-methylbutyryl-CoA dehyd. activity	GO:0047015	3.40E-05	1.10E+01

nutrient reservoir activity	GO:0045735	3.40E-05	9.30E+00

structural constituent of cell wall	GO:0005199	3.60E-05	7.90E+00

2-nitropropane dioxygenase activity	GO:0018580	4.10E-05	1.40E+01

adenylate cyclase activity	GO:0004016	6.00E-05	9.50E+00

^b ^beta-lactam antibiotic catabolic process	GO:0030655	6.70E-05	1.40E+01

DNA primase activity	GO:0003896	8.40E-05	1.10E+01

cyclic nucleotide biosynthetic process	GO:0009190	8.50E-05	9.80E+00

iron ion transport	GO:0006826	8.70E-05	1.20E+01

di-, tri-valent inorganic cation transmembrane transporter activity	GO:0015082	9.00E-05	6.80E+00

phosphorus-oxygen lyase activity	GO:0016849	1.60E-04	9.50E+00

limonene-1,2-epoxide hydrolase activity	GO:0018744	1.60E-04	7.80E+00

^c ^fatty acid metabolic process	GO:0006631	1.70E-04	8.40E+00

sirohydrochlorin cobaltochelatase activity	GO:0016852	1.80E-04	1.50E+01

intracellular signaling cascade	GO:0007242	2.00E-04	8.80E+00

^c ^enoyl-CoA hydratase activity	GO:0004300	2.30E-04	1.30E+01

di-, tri-valent inorganic cation transport	GO:0015674	2.40E-04	6.20E+00

^c ^acyl-CoA dehydrogenase activity	GO:0003995	2.60E-04	9.10E+00

catechol O-methyltransferase activity	GO:0016206	3.20E-04	1.50E+01

^a^Bonferroni-corrected p-value calculated from T-test

^b ^pathogenicity or survival within the host

^c ^Lipid metabolism

^d ^Cofactor biosynthesis

^e ^unknown function

### Substantial expansion of known pathogenicity and lipid metabolism genes

Despite the smaller genome sizes present in the pathogenic Mycobacteria, and the resulting background of orthogroup loss in the evolution towards pathogens, we observe significant expansions in certain gene families in the pathogenic Mycobacteria and the *Mtb *complex relative to non-pathogenic relatives. We also observe evidence for selection in certain families on branches leading to the pathogenic Mycobacteria, the *Mtb *complex, and the soil-dwelling Mycobacteria.

As expected, many genes known to be related to pathogenicity or antigenic variability are among the groups most expanded in the *Mtb *clade relative to soil dwelling Mycobacteria as well as being among the categories with the most variability in copy number in their category-level profiles overall, including toxin-antitoxin genes, genes containing PE (Pro-Glu) and PPE (Pro-Pro-Glu) domains, MCE (Mammalian Cell Entry) genes, genes involved in the synthesis of the mycolic acid coat, Esx genes, and gene involved in antibiotic resistance. Complete results for all groupings are available on our **Supplementary Information **website. Below we focus on specific additional families showing noteworthy expansions and trends.

The single most significant trend in our analysis of protein family evolution is that genes related to lipid metabolism are greatly expanded across all Mycobacteria and related organisms, consistent with previous observations [2,31] (Table 5). Our analysis extends these previous observations by identifying the emergence of this expansion in lipid metabolism genes as occurring at the root node of the Mycobacteria and *Rhodococcus *(Figure 3).

### Particular expansion of saturated fatty acid metabolism and lipid synthesis genes in pathogenic Mycobacteria

**Figure 4:**
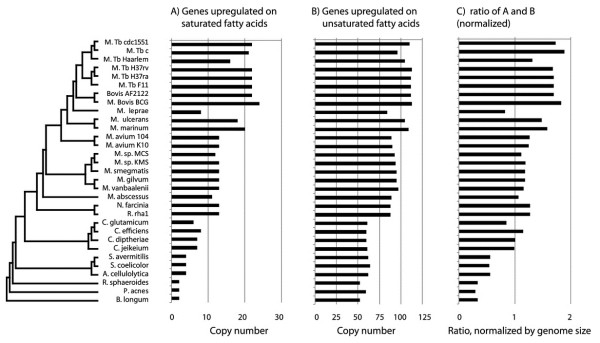
**Evolution of genes upregulated when grown on saturated or unsaturated fatty acids**. Genes upregulated by at least 1.5 standard deviations are indicated here. a) Genes expressed under palmitic acid but not oleic or linoleic (genes expressed in saturated fatty acid conditions). b) Genes expressed under linoleic or oleic acid but not palmitic (genes expressed under unsaturated fatty acid conditions). c) The ratio of the phylogenetic profiles for genes expressed under palmitic and linoleic acid, normalized by genome size.

Genes predicted to be involved in the metabolism of saturated fatty acids are more expanded than those involved in the metabolism of unsaturated fatty acids. Using a compendium of microarray expression experiments (**Methods**), we compiled a list of genes upregulated in the presence of different fatty acid sources. We found that genes upregulated under unsaturated conditions have more uniform phylogenetic profiles, while those upregulated under saturated conditions, cholesterol or ceramide have expanded through duplications in pathogenic Mycobacteria (Figure 4). Saturated fatty acids and cholesterol are more prevalent in an animal host than in the soil, which contains mostly unsaturated fatty acid from plant inputs. Since it is believed that *Mtb *uses cholesterol as a carbon source within the host [32], this could reflect an adaptation to the host environment. Consistent with our observations in host-adapted Mycobacteria, *Desulfovibrio desulfuricans *intestinal strains contain a higher ratio of saturated to unsaturated fatty acids than soil strains of *Desulfovibrio desulfuricans *[33].

Our analysis also reveals differences in evolutionary profiles between genes predicted to be involved in catabolism and anabolism of lipids. Both sets of genes are expanded in soil-dwelling and pathogenic Mycobacteria, but lipid synthesis genes are additionally expanded in pathogens relative to soil dwellers. General lipid synthesis genes are expanded across the Mycobacteria, but certain groups of lipid synthesis genes, including those related to cell wall synthesis, are further expanded in the *Mtb *complex (see Supplementary Information). In pathogenic Mycobacteria, the waxy mycolic acid coat helps evade the host immune system [34]. Consistent with this, we see categories related to mycolic acid synthesis showing up among the most non-uniform categories, highly expanded in the *Mtb *complex (see Supplementary Information).

In contrast, some lipid degradation gene families are more expanded in the soil-dwelling Mycobacteria than in the pathogens (**Supplementary Data**). The soil-dwellers have the unusual ability to degrade a vast array of compounds, including diverse lipids.

### Positive selection of lipid metabolism genes

In addition to gene family expansions, we observe evidence for selection on the coding sequence of lipid metabolism genes. In our d_N_/d_S _calculations, we observe enrichment for positive selection in lipid degradation genes on the branch leading to the pathogenic Mycobacteria (Additional file 1: Table S2). For example, Rv2524c, the multifunctional FAS-I polypeptide utilized during *de novo *fatty synthesis [35], has the second highest d_N_/d_S _value on this branch. Additional lipid metabolism genes with elevated d_N_/d_S _values include 15 genes predicted to be involved in the β-oxidation pathway of fatty acid degradation: seven *fadE *(acyl coA dehydrogenase) genes, three *fadD *(fatty acid CoA ligase) genes, two *fadB *(NADPH quinone oxidoreductase/3-hydroxybutyryl-CoA dehydrogenase) genes, one *fadA *(acetyl-CoA acyltransferase) gene, and two *echA *(enoyl-CoA hydratase) genes. Hence, we observe expansions of lipid biosynthesis genes, as well as observing evidence for positive selection acting on genes within the β-oxidation pathway. Both the lipid biosynthesis and lipid degradation pathways are specialized within the pathogenic Mycobacteria. This expansion could possibly benefit the pathogen in a manner to accommodate production and modification of cell wall lipids involved in manipulation of host immune response. The lipid degradation is particularly beneficial for the long term survival of the pathogen metabolizing host lipids encountered during infection.

### Coordinated evolution of lipid metabolism genes and the regulator KstR

KstR is a transcription factor known to be involved in lipid and cholesterol degradation [36,37]. It has been recently shown that *Mtb *uses cholesterol as a carbon source within the host [32]. Strikingly, KstR exhibits an evolutionary history that parallels the expansion of lipid metabolism genes in the Mycobacteria, and displays a singular conservation in its regulatory binding sites.

KstR appears to have evolved at the last common ancestor of the Mycobacteria and Rhodococcus. In all Mycobacteria analyzed (except *M. leprae*), *Rhodococcus*, and *Nocardia *there is one highly conserved ortholog of the *KstR *gene. However, in organisms more distantly related to *Mtb*, the *KstR *gene is not present in a single copy. Rather, 2-3 paralogs of *KstR *are present in these more distantly related organisms, as well as the environmental Mycobacteria, including *M. ulcerans, M. avium 104, M. sp. MCS, M. sp. KMS*, and *M. vanbaalenii *(Additional file 2: Figure S1). Remarkably, these paralogs of KstR are all absent in the pathogenic Mycobacteria. Thus, coincident with the expansion in lipid metabolism genes described above, the *KstR *gene appears to have emerged through gene duplication within the existing gene family of tetR-like transcriptional regulators at the last common ancestor of *Mycobacteria *and *Rhodococcus*. All other members of this gene family were subsequently lost in the *Mtb *complex, while the KstR protein was maintained and underwent limited sequence divergence.

There is another homolog to KstR found in *Mtb H37Rv *(*Rv3557c*) that has previously been reported to also be involved in cholesterol metabolism, named KstR2 [38]. However, KstR is much more similar to the other members of the Mycobacterial tetR family discussed above than it is to KstR2. KstR2 is categorized into a separate orthogroup (orthogroup 32655) and is more distantly related to KstR.

The high sequence conservation of the KstR transcription factor is mirrored in the conservation of KstR binding sites across numerous promoters. KstR binding sites are known to be highly conserved across the Mycobacteria, out to *Rhodococcus *and *Nocardia *[36]. These sites are conserved in both sequence and position within their respective promoters. In our analysis, both in searches using known transcription factor binding motifs, as well as in our *de novo *motif searches, a subset of KstR binding sites are the most conserved transcription factor motifs observed. They are also among the most conserved of any noncoding sequence we identified. The conservation of the KstR gene and binding sites, the emergence of KstR at the ancestor of *Rhodococcus *and the Mycobacteria, and the loss of KstR paralogs within the pathogenic Mycobacteria, suggests that this transcription factor and its evolving regulon have played an important role in the expansion of lipid metabolism and its adaptation to pathogenicity in *Mtb*.

### Positive selection of DNA repair genes

*Mtb*, as well as non-tuberculous Mycobacteria, differ from other bacteria in several key respects of DNA repair [39-42]. Within the host, *Mtb *must combat damage to its DNA from macrophage-generated reactive oxygen and nitrogen intermediates. The mechanisms by which this is accomplished are not fully understood [43,44]. Although genes implicated in DNA repair have not expanded in the *Mtb *lineage, we note that the set of genes showing positive selection on the *Mtb *lineage in our d_N_/d_S _analysis is enriched for genes involved in the COG category for DNA replication, recombination, and repair (Additional file 1: Table S2). Several of the genes in this set with highest d_N_/d_S _values are known DNA repair genes (including *recA, recB*, and *dnaE2*), and several additional genes are helicases (*dnaB, helZ*, and *gyrB*).

Interestingly, we observe that *recA *has the highest d_N_/d_S _score of all the genes in *Mtb *on the branch leading to the *Mtb *complex, and *recB *also has a very high score. Mycobacteria lack a mutSL-based mismatch repair (MMR) system [42], and it is believed that *recA *may be involved in compensating pathways. *dnaE2 *(DNA polymerase III) also has one of the highest d_N_/d_S _values on the branch leading to *Mtb*, and both *dnaE1 *(DNA polymerase III) and *dnaE2 *show evidence of selection on the branch leading to the pathogenic Mycobacteria. In *Mtb*, damage-induced base-substitution mutagenesis is dependent on *dnaE2*. Loss of *dnaE2 *activity renders *Mtb *hypersensitive to DNA damage, eliminates induced mutagenesis, attenuates virulence, and reduces the frequency of drug resistance in vivo [39,45]. *dnaE1 *provides essential, high-fidelity replicative polymerase function [39], and is expressed in response to DNA damage, along with *dnaE2 *and *recA *[39,45].

We also observe positive selection for *dinX *(DNA polymerase IV) on the branch leading to the pathogenic Mycobacteria (branch-site model) in our d_N_/d_S _analysis (see Supplementary Information website). Most organisms use specialized DNA polymerases that are able to catalyze translesion synthesis (TLS) across sites of damage, including the *dinB *group of Y family polymerases. There are two *dinB*-family polymerases in *Mtb *(*dinX *and *dinP*). Unlike in other bacteria, *dinX *and *dinP *expression are not dependent on *recA*, the SOS response, or the presence of DNA damage, and could therefore serve a novel yet uncharacterized role in *Mtb *[46-49].

### Expansion of pterin cofactors

Genes involved in the first steps of pterin cofactor (a component of the molybdenum cofactor) biosynthesis are known to be expanded in the *Mtb *complex [50]. Molybdenum cofactor-requiring enzymes (such as xanthine oxidase and aldehyde oxidase) could have physiological functions in the metabolism of reactive oxygen species during stress response [51]. Molybdenum cofactor is an efficient catalyst in oxygen-transfer reactions, can be used in anaerobic respiration, and can catalyze redox reactions in carbon, nitrogen, and sulfur metabolism. Recently, genes related to molybdenum cofactor protein synthesis have been shown to be upregulated under conditions of stress in *Mtb *[52]. Molybdenum cofactor biosynthesis has been previously linked to pathogenesis. The regulator of the *moa1 *locus, *MoaR1*, was identified as having a SNP in *M. bovis BCG*, but not in virulent *M. bovis *or *Mtb *[53]. In addition, *moa3 *is present with varying frequency in the RD1 region, which is absent in *M. bovis BCG*, of pathogenic strains [54].

In agreement with previous observations of expansions of molybdopterin biosynthesis genes, we observe five protein domains related to pterin cofactor biosynthesis among the top protein domains expanded in the *Mtb *complex compared to the non-pathogenic Mycobacteria (Table 2, -"d"). Among the top GO terms expanded in the *Mtb *clade relative to the soil dwellers (Table 3), there are also several groups involved in pterin and molybdopterin biosynthesis. Some of these gene copies (the *moa1 *locus) are believed to have been acquired by lateral gene transfer on the branch leading to the *Mtb *complex [10,50].

We also observe evidence for selection on molybdenum-related genes in our d_N_/d_S _data. On the branch leading to the pathogenic Mycobacteria, several orthogroups with high log likelihood scores when testing for selection are related to molybdenum (see Supplementary Information website). The orthogroup containing BisC (biotin sulfoxide reductase, a molybdoenzyme), as well as the orthogroup containing ModA (an ABC-family molybdate transporter), are among those with the highest d_N_/d_S _values on the branch leading to the pathogens. *MoaB2 *is one of the highest-scoring genes on all three branches tested.

### Expansions of genes of unknown function in *Mtb *clade

There are also many categories of unknown function that are greatly expanded in the *Mtb *clade relative to the non-pathogenic Mycobacteria (Tables 2 and 3, red). For example, *Rv0918 *(in the Pfam group of unknown function PF08681) was found in a genetic screen that facilitates isolation of mutants defective in arresting the maturation of phagosomes [55], helping *Mtb *to survive within host cells. PF07161 contains four lipoproteins (LprF, LprG, LprA, LppX). *LprG *and *LppX *were found to be in vivo essential genes by TraSH analysis [56].

### Detection of conserved noncoding sequences

Sequence conservation - or phylogenetic footprinting - provides a powerful approach for identifying potential functional noncoding sequences, and has been used in a variety of eukaryotic and prokaryotic organisms to identify protein coding genes, noncoding RNAs, and regulatory elements [57,58]. For optimal power, the organisms being analyzed must be sufficiently distant such that non-functional elements have diverged, but not so distant such that functional elements have evolved or re-arranged. Organisms within the *Mtb *complex are all highly similar at the sequence level, and thus by themselves do not allow for effective phylogenetic footprinting. By leveraging the evolutionary similarity of the most distantly related Mycobacteria and Actinomycetes, we gained additional power to allow us to detect functional sequences under purifying selection, albeit only those shared by at least a majority of Mycobacteria. We used this approach to predict two classes of conserved noncoding sequences: small noncoding RNAs and transcription factor binding motifs.

### Novel putative conserved small noncoding RNAs in Mycobacteria

Small noncoding RNAs (sRNAs) have been shown to play a role in regulating gene expression in numerous bacterial species [59], including *Streptococcus *[60,61]. Yet only recently were sRNAs reported in Mycobacteria [60,62]. Using a combination of direct isolation of small RNAs, and validation by Northern blotting and 5' and 3' RACE transcript mapping, Arnvig and Young [62] first described nine sRNAs in *Mtb*. Subsequently, DiChiara et al. [63] describe 34 small RNAs in *M. bovis *BCG, of which many were conserved in both *Mtb *and *M. smegmatis*.

**Figure 5:**
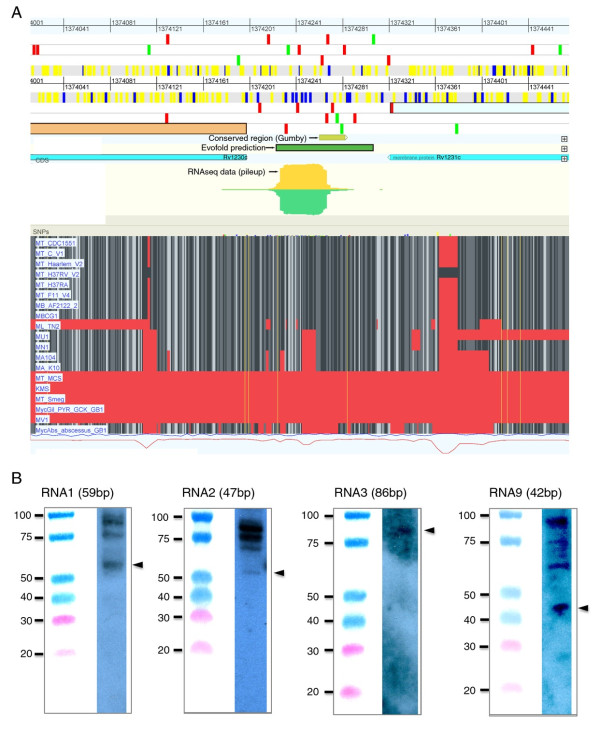
**New predicted RNAs**. a) An example of a new predicted RNA. This is the RNA2 in Table 6. This figure shows a screenshot from the GenomeView Browser [64]. The light blue bars show the coding regions (*Rv1230c *and *Rv1231*); the tan bar shows the conserved region predicted by Gumby [65]; and the green bar shows the region predicted to fold by Evofold [66]. The yellow and green plots in the center show the RNA-seq data. Green signifies reads from the negative strand, and yellow shows the total reads (positive and negative strands). The multiple alignment is shown on the bottom (darker grey signifies a higher degree of conservation; red signifies no alignment at that position). You can see that this predicted RNA region is conserved through *M. avium*. The rulers at the top show the gene structure. Small red squares show where stop codons are present all six reading frames, indicating that this intergenic region is unlikely to be a protein-coding region missed in the annotation. b) Northern blots validating four of the new, predicted small RNAs (RNA1, RNA2, RNA3, and RNA9 in Table 6).

To build on these results, we used a combination of comparative genomics, RNA-seq, and experimental validation by Northern blotting to identify additional sRNAs conserved among the Mycobacteria (**Methods**). Our computational results provide evidence for 50 conserved small RNAs in *Mtb *that have not been previously reported. It is likely that additional conserved regions are expressed under other diverse conditions. Figure 5a shows the expression and conservation map for one of our predicted RNAs in the GenomeView Browser [64]. Table 6 shows a listing of the top 12 candidate RNAs. To verify a subset of these candidate small RNAs, we used Northern blot analysis on four of the top predicted regions (**Methods**). The results (Figure 5b) show signals corresponding to small RNAs from each of four candidates (Table 6, labeled 1, 2, 3, and 9). All transcripts were near the expected size, or slightly larger. Full-length gels are provided in Additional file 3: Figure S2. Consistent with previous work, the majority of small RNAs were seen as more than one size transcript [62]. This suggests that small RNAs might be generated by processing of larger transcripts. In the RNA-seq data, there are longer "tails" extending outside of the main peak that corresponds to the RNA prediction--different length RNAs could be responsible for the additional bands of higher mass.

**Table 6:** Top 12 predicted RNAs, ranked by their RPKM score

	Conserved region in *Mtb H37Rv*^1^								Region in *M. Smegmatis*^3^			
**id**	**start**	**Orient-ation^5^**	**length**	**# reads**	**RPKM**	**Evo-fold?^2^**	**Genes flanking the intergenic region**		**Start**	**Stop**	**# reads**	**RPKM**

RNA1^4^	1612987	--+	58	1013	69526	Y	*Rv1435c *(secreted protein)	*Rv1436 *(GAPDH)	756567	756626	100	626

RNA2^4^	1374224	---	46	567	30071	Y	*Rv1230c *(membrane prot.)	*Rv1231c *(membrane prot.)	-	-		-

RNA3^4^	1393055	---	85	111	9251	Y	*Rv1248c *(sucA)	*Rv1249c *(membrane prot.)	5147110	5147242	173	489

RNA4	483829	-++	49	55	2139	Y	*Rv0403c *(membrane prot. mmpS1)	*Rv0404 *(fadD30)	-	-		-

RNA5	1200514	-++	93	81	2055	Y	*Rv1075c *(exported protein)	*Rv1076 *(lipase lipU)	5363400	5363474	10	50

RNA6	987053	-++	92	174	6767	Y	*Rv0887c *(cons. Hypo. prot)	*Rv0888 *(hyp. Exported prot.)	-	-		-

RNA7	1810184	+++	44	517	9280	Y	*Rv1610 *(cons. membrane protein)	*Rv1611 *(trpC)	3296996	3297034	291	2804

RNA8	3587635	-++	56	83	6917	N	*Rv3210c *(cons. hypo. prot.)	*Rv3211 *(rhlE)	2009575	2009665	6	25

RNA9^4^	4224925	--+	41	502	24405	N	*Rv3778c *(aminotransf.)	*Rv3779 *(membrane prot.)	6420618	6420674	36	237

RNA10	659351	+++	39	58	1829	Y	*Rv0567 *(methyltransferase)	*Rv0568 *(cyp135B1)	-	-		-

RNA11	1794708	-++	48	375	18231	Y	*Rv1593c *(cons. hypo. prot)	*Rv1594 *(nadA)	3277552	3277599	70	548

RNA12	2447526	---	74	332	9684	Y	*Rv2185c *(cons. hypo. prot)	*Rv2186c *(cons. hypo. prot)	4335086	4335137	111	802

^1^Conserved intergenic regions determined by Gumby.

^2^Indicates whether this region is predicted to fold by Evofold.

^3^Region in *M. smegmatis *that aligns with the conserved region in *Mtb*, and its corresponding RPKM value.

^4^Tested experimentally

^5^Orientation relative to neighboring genes. The first and last characters give the strands of the flanking genes; the middle character gives the strand for the predicted RNA.

### Conserved *cis*-regulatory motifs in Mycobacteria

Few transcription factor binding motifs have been identified in *Mtb*. Transcription factors for which binding motifs have been identified include KstR [36], DosR [67], IdeR [68], ZurB [69], Crp [70], CsoR [71], FurA [72], MprAB [73], and Acr [74]. Because of the limited knowledge of transcriptional regulation in *Mtb*, we searched for additional motifs computationally. We combined comparative sequence analysis with microarray data to identify a large number of motifs conserved in Mycobacteria.

We clustered microarray data contained in the TB database [75] and searched for upstream regulatory motifs shared in the upstream regions of the resulting clusters using AlignACE (**Methods**). Because of significant noise in the results, we used a set of stringent filters, including a requirement that candidate motifs be highly conserved. 37 motif instances passed our stringent filters (Table 7, **Methods**). 14 of the top 37 (38%) motif instances correspond to cases of known *Mtb *motifs (several known *Mtb *motifs were found more than once, in different clusters, or in clusters with different size parameters). In contrast, none of the top motifs showed similarity only to known *E. coli *or *Corynebacteria *motifs. Within these top motifs, we were able to identify four of the nine known *Mtb *motifs (DosR, IdeR, KstR, and ZurB).

**Table 7:** Motifs passing our set of stringent filters, ranked by their degree of conservation

k^a^	Cluster #	Motif	MAP score^b^	Specificity^c^	Known^d^	Palind-romicity^e^	Conser-vation^f^	Motif Logo^g^
100	71	1	65.6	1.8E-29	KstR	0.93	46	

250	179	1	88.6	4.8E-26	KstR	0.71	43	

50	35	1	87.4	1.8E-19	KstR	0.79	40	

200	182	1	94.2	5.7E-28	KstR	0.80	36	

100	73	2	31.3	1.5E-32	IdeR	0.85	30	

100	49	15	16.3	2.8E-18	KstR	0.71	29	

250	112	1	25.5	2.1E-25	DosR	0.76	21	

100	80	1	15.0	2.2E-14		0.92	21	

50	29	1	15.0	2.2E-14		0.92	21	**X **

50	23	31	21.7	6.7E-24		0.71	18	

200	47	64	7.2	1.2E-15		0.75	16	

250	87	1	22.1	1.5E-13	ZurB	0.94	16	

200	6	1	16.3	2.0E-12	IdeR	0.70	16	

200	184	19	12.1	4.7E-13		0.75	16	

250	123	18	14.5	3.7E-11		0.82	16	

250	224	1	26.9	6.2E-24	DosR	0.73	15	

200	46	3	19.8	1.6E-12		0.78	15	

100	5	25	12.4	3.8E-15		0.71	14	

200	120	62	15.3	2.0E-14		0.74	14	

200	71	9	16.6	2.3E-16		0.75	14	

200	195	1	25.4	7.2E-26	DosR	0.76	14	

50	48	1	68.8	6.4E-35	DosR	0.86	13	

100	74	1	66.9	3.6E-34	DosR	0.88	13	

50	23	12	26.8	5.0E-22		0.73	13	

250	89	1	46.636	4.0E-20		0.75	12	

100	81	84	6.4	2.3E-16		0.71	12	

250	92	6	5.7	3.7E-17	DosR	0.73	12	

100	52	91	9.7	1.5E-18		0.76	11	

200	91	1	47.1	4.7E-20		0.70	10	

50	36	43	34.8	4.6E-30		0.72	10	

100	42	4	15.3	2.8E-17		0.70	10	

200	80	9	8.5	4.0E-13		0.72	10	

50	43	7	15.7	8.7E-12		0.72	10	

50	36	21	48.5	5.3E-12		0.71	10	

200	26	17	7.5	2.1E-12		0.70	9	

50	6	61	6.3	1.2E-11		0.73	9	

50	11	43	13.7	1.4E-11		0.72	14	

^a^k indicates the value of k in the k-means clustering process (50, 100, 200, or 250)

^b^MAP score indicates the AlignACE MAP score [76]

^c^Specificity score [77]

^d^CompareACE score ≥ 0.7 to the alignment for this known motif

^e^CompareACE score to its reverse complement

^f^number of ScanACE hits in the genome that are conserved in ≥ 8 genomes

^g^sequence logo [78]

As described above, the KstR motif shows a much stronger signal, in terms of both conservation and information content, than any of the other motifs (top of the ranked conservation list, Table 7). Based on the distribution of highly conserved predicted motif instances for KstR across the genome, we predict a more general role for KstR in lipid metabolism. We see KstR motif instances near many other lipid genes not related to cholesterol degradation, in support of the view that KstR is a more general lipid regulator controlling a large regulon [36].

**Figure 6:**
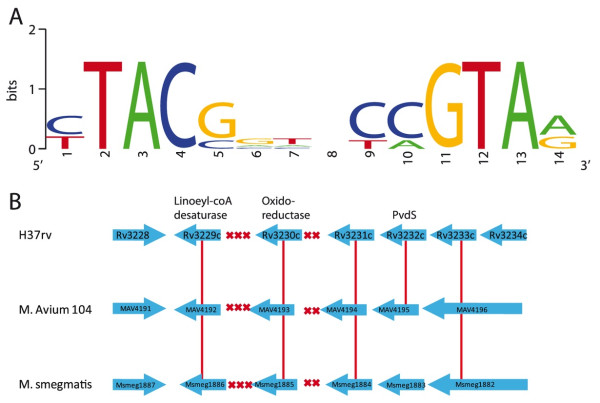
**New predicted motif with binding sites upstream of fatty acid-related genes**. a) Motif logo. b) Conserved binding site locations for this new motif are marked with red x's. Red lines indicate orthologous relationships between genes in *Mtb H37Rv, M. avium 104*, and *M. smegmatis*.

One of the most interesting new motif candidates that shows up in our analysis is a conserved palindromic motif, consisting of a highly conserved TAC... GTA separated by 6 bp of less well conserved sequence (marked with an X in Table 7) that is found in clusters of 2-3 closely spaced sites upstream of several genes related to fatty acid metabolism (Figure 6). There is a cluster of 3 evenly spaced sites upstream of *Rv3229c *(linoeyl-coA desaturase), a cluster of 2 sites upstream of the adjacent Rv3230c (oxidoreductase), and a cluster of 3 sites upstream of *Rv2524c *(fatty acid synthase). This is the second highest-scoring new motif identified (Table 7). This motif shows up as one of the top motifs associated with the clusters of genes upregulated under saturated fatty acid conditions (specifically palmitate).

## Conclusion

To better understand *Mtb*, we performed a comparative analysis of 31 organisms from the Tuberculosis Database. We studied the evolution of protein families and metabolic pathways, looked for proteins with evidence of selection, and searched for new noncoding RNAs and transcription factor binding site motifs.

The most striking features of our analysis are related to lipid metabolism and its regulation. In addition to observing a general expansion of lipid metabolism genes in the Mycobacteria and *Rhodococcus*, we observe increased expansions of genes related to saturated fatty acid metabolism in the pathogenic Mycobacteria compared to the soil-dwelling Mycobacteria. We also note differences in evolutionary profiles for catabolic and anabolic lipid metabolism genes, and evidence for positive selection in lipid metabolism genes. The *cis*-regulatory elements bound by the KstR protein, a known regulator of lipid/cholesterol metabolism, are among the strongest, most highly conserved noncoding signals across the Mycobacteria. Both KstR and its binding sites are highly conserved, appearing at the last common ancestor between *Rhodococcus *and the Mycobacteria.

Within our set of organisms, we examine the evolution of pathogenicity, moving from the soil-dwelling Mycobacteria up to the intracellular parasites of the *Mtb *complex. We see expansions of many known gene families related to pathogenicity (PE/PPE genes, antibiotic resistance genes, genes involved in the synthesis of the mycolic acid coat, MCE genes, and Esx genes). By similarity of phylogenetic profiles, we can predict likely candidates for novel gene families related to pathogenicity. For example, we see similar expansions in gene families related to biosynthesis of molybdopterin. We further observe evidence of positive selection on molybdenum-related genes, providing further support for the importance of molybdenum in these pathogens. On the branch leading to the pathogenic Mycobacteria, we also observe evidence for positive selection in genes related to replication, recombination, and repair. It is possible that these DNA repair-related processes give the pathogenic Mycobacteria an advantage when dealing with the assault on its DNA by macrophage-generated reactive oxygen and nitrogen intermediates.

Our whole-genome alignments, coupled with RNA-seq and microarray data, allowed us to predict novel noncoding features, including small RNAs (four of which we have validated experimentally), and potential transcription factor binding sites.

The main forces driving genome evolution in prokaryotes include gene genesis, lateral gene transfer, and gene loss. Our analysis of protein evolution using SYNERGY does not examine whether orthogroups appearing have arisen by lateral gene transfer or by gene genesis involving duplication and divergence from other orthogroups. A detailed comparison to categorize these orthogroup appearances according to lateral or vertical gene transfer is beyond the scope of this study, but other studies indicate that lateral gene transfer has played a significant role in Mycobacterial evolution and the evolution of pathogenesis [79-83].

A recent paper suggests that the Mycobacterial genome has been shaped by a biphasic process involving gene acquisition (including lateral gene transfer) and duplications followed by gene loss [79]. Other studies report numerous genes, including a large number involved in lipid metabolism, that have been acquired by horizontal gene transfer at different phylogenetic strata and have led to the emergence of pathogenesis in *Mtb *[80,81]. Previous studies indicate a possible more ancient lateral gene transfer of fatty acid biosynthesis genes from α-proteobacteria to actinobacteria [84]. However, genetic studies show that the *Mtb *complex and pathogenic Mycobacteria do not exchange genetic material frequently [85,86], so there is limited lateral gene transfer within the *Mtb *complex.

We are currently performing high-throughput Chromatin Immunoprecipitation (ChIP)-Seq experiments in several different Mycobacteria, including *Mtb, M. smegmatis*, and *M. vanbaalenii*[87]. We plan to integrate the information obtained from our comparative analysis with data coming from these high-throughput experiments, as well as other 'omic datasets, using a systems biology approach. This will enable construction of gene regulatory networks for *Mtb*, and examination of their evolution across species.

## Methods

### Genome sequences

The 31 organisms used in our analysis are described in Table 1. These genome sequences are all contained in the TB database (TBDB) [75]. The three unpublished sequences generated at the Broad Institute (*M. tuberculosis F11, M. tuberculosis Haarlem*, and *M. tuberculosis C*) are high-quality genome sequences. *M. tuberculosis F11 *and *M. tuberculosis Haarlem *are finished, and *M. tuberculosis C *has 6.7× coverage and 4 scaffolds. The Broad Institute sequencing read pipeline interacts with the sample management system to ensure the read is associated with the correct sample. Vector identification, length checks and quality clipping were performed on all reads. Contamination checks and organism checks were also performed using a kmer-based algorithm that can compare sequence to a profile from any organism.

### Defining protein families and constructing phylogenetic trees

The SYNERGY algorithm [27,28] was applied to the 31 genomes in Table 1. SYNERGY organizes groups of genes across organisms into orthogroups, or groups of orthologs and paralogs, which consist of all the genes descended from a single ancestral gene in the species' last common ancestor. SYNERGY also associates orthogroups with a gene tree, from which we can derive an "extended phylogenetic profile", showing the gene copy number in each extant organism and at each ancestral node. Importantly, by reconciling an organism tree with each gene tree, SYNERGY provides an evolutionary scenario for each gene tree predicting where all losses, gains, and duplications occurred in its evolution. These lists of losses, gains, and duplications contain actual evolutionary events, as well as artifacts caused by genes that could not be properly categorized by SYNERGY. However, we observe that SYNERGY is effective at properly categorizing genes into orthogroups, and the SYNERGY orthogroups were very useful in our analysis. Analysis of the 31 genomes resulted in a total of 32,505 orthogroups, including those containing single genes from only a single genome (below). There were 177 "uniform" (1:1:1:1...) orthogroups representative of some of the most conserved and indispensible housekeeping genes. Additional file 4: Figure S3 summarizes the SYNERGY orthogroups.

We started running SYNERGY using an initial phylogenetic tree generated using orthologs based on bidirectional best BLAST hits. The list of uniform orthogroups from the first SYNERGY run was used to construct a refined phylogenetic tree. SYNERGY was then re-run using the refined phylogenetic trees. To generate our final phylogenetic tree, the final set of 177 31-way orthologs (31-way uniform orthogroups from the SYNERGY analysis) were aligned according to their nucleotide sequences with CLUSTALW [88] and concatenated, distances were computed with Phylip's DNADIST algorithm [89], and Phylip's FITCH algorithm was used to create the tree.

Because of the similarity of the genomes within the *Mtb *complex, we were not able to resolve the phylogeny using only these 177 proteins that are uniform across all 31 organisms. In order to better resolve the tree within the *Mtb *cluster, we computed a separate tree using 1747 orthogroups that are uniform across the *Mtb *cluster and *M. ulcerans*, which we used as an outgroup. Using this expanded gene set, we were able to resolve the tree for the *Mtb *cluster.

Bootstrap analysis was performed to validate tree topologies. Phylip's SEQBOOT was used to create 1000 bootstrap input replicates for each tree. Phylip's CONSENSE was used to obtain a bootstrap tree (Additional file 5: Figure S4)

### Metabolic pathways and functional groups

EFICAZ [90] was used to assign EC numbers for proteins in all 31 organisms. Metabolic pathways were constructed in Biocyc [91,92]. An orthogroup was considered to be part of a metabolic pathway if any of its component genes had been identified as part of that pathway using this pipeline.

We obtained the Gene Ontology (GO) [29] and GO Slim terms for each of the 31 organisms using BLAST2GO [93]. PFAM assignments [30] were taken from http://www.tbdb.org[75]. An orthogroup was associated with a GO, GO Slim, or PFAM descriptor if greater than half of its protein members were associated with that descriptor.

For each node in the phylogenetic tree, we tabulated orthogroups lost, gained, or duplicated. Using GO terms, GO Slim terms, and PFAM domain groupings with less than 500 members, we calculated over-representations within losses, gains, and duplications each of these groupings at each node using the hypergeometric test. A complete summary of gains, losses, and duplications for all nodes in the phylogenetic tree is available on our supplementary information website.

### Phylogenetic profiles

Extended phylogenetic profiles for each category (metabolic pathways, GO terms, GO Slim terms and PFAM categories) were obtained from SYNERGY output by summing the phylogenetic profiles from their component orthogroups. We define a category-level phylogenetic profile as the sum of its component orthogroup-level phylogenetic profiles. The evolution of each of these categories can be quickly visualized on our website. Since genes with the same phylogenetic profile can be linked functionally [94], the webpage for each category contains a link to other categories with similar phylogenetic profiles (**Methods**). Categories with the most similar profiles were obtained by calculating Euclidean distances to all other profiles.

Instances of expanded or missing pathways across the 31 organisms will have non-uniform pathway-level phylogenetic profiles. Thus we tabulated the number of genes in each genome for each category, and automatically searched for gene categories whose copy number (normalized for genome size) had the most non-uniform distribution across the 31 organisms in order to identify the most significant examples of expansions or losses. To identify categories with bimodal properties (such as a categories with a loss or a large expansion on only certain branches of the phylogenetic tree), we clustered each profile into two groups and looked for the pathways with the greatest separation between the two clusters. We used k-means (k = 2) to cluster the profile vectors, and compared the intra- and inter-cluster point-to-centroid distances to find the clusters with the greatest separation. We ranked categories by this separation to find bimodal categories. We further select those that have at least five organisms in the smallest of the two clusters, and an average of at least five genes per genome. P-values are calculated from a T-test between the values for the two groups, with Bonferroni correction applied. In our Supplementary Information website we list those categories with p < 0.05, ranked by the difference between their inter- to intra-centroid distances. When we select the metabolic pathways, PFAM domains, and GO terms with the most non-uniform category-level phylogenetic profiles overall, we find that many of the top categories are lipid metabolism-related categories expanded in the Mycobacteria.

We also measured the similarity between evolutionary profiles to find the PFAM categories and GO terms with the biggest difference between pre-defined sets of organisms. For example, we compared both the *Mtb *complex and a group consisting of other pathogenic Mycobacteria to the set of soil-dwelling Mycobacteria in order to examine the evolution of soil-dwelling, free-living Mycobacteria into more pathogenic Mycobacteria that require a host to survive. We used the following categories:

1. All Mycobacteria (excluding *M. leprae *because of its massive gene loss).

2. All non-Mycobacteria in our set (excluding *Nocardia *and *Rhodococcus *because of their similarity to Mycobacteria)

3. *Mtb *complex (8 organisms)

4. Other pathogenic Mycobacteria (*M. ulcerans, M. avium 104, M. avium K10, M. marinum*).

5. Soil-dwelling Mycobacteria that do not require a host (*M. sp. MCS, M sp. KMS, M. smegmatis, M. vanbaalenii, M. abscessus, M. gilvum*).

6. *R. jostii RHA1 *and *N. farcinia*

We calculated differences between two sets of organisms exactly as we calculated distances between clusters (above). However, rather than using different clusters of organisms determined by k-means clustering, we used these pre-defined clusters of organisms. We looked at distances between the following sets of organisms: 1-2, 3-4, 3-5, 3-6, 4-5, 4-6, 5-6. For each PFAM domain or GO term represented in at least two organisms in these pairings, we calculated p-values for the differences between the profile values by T-test (Bonferroni-corrected by the number of PFAM domains represented in that set of organisms) and computed inter-and intra-centroid distances (as described in the above paragraph). We compiled lists of those that are most expanded and a list of those most contracted across these pairings. On our website we have included complete lists of PFAM categories, including those that do not make the strict Bonferroni-corrected p-value cutoff. Many potentially interesting expansions do not make the overly conservative Bonferroni-corrected p-value cutoff [95,96].

### Motif discovery

Using a compendium of 946 microarray experiments from the TB database [75], we used several different clustering methods to generate predicted regulons. We searched the upstream regions of these regulons for shared transcriptional regulatory motifs. We clustered microarray data by hierarchical and k-means clustering. Because real regulons can be of varying sizes, we performed k-means with k = 50, 100, 200, and 250, then used all the resulting clusters for further analysis. We found that the clusters obtained from hierarchical clustering were not very useful because their size distribution did not approximate that of real regulons as well as those from k-means; therefore we did not analyze clusters from hierarchical clustering further.

We used AlignACE [97] to search the upstream regions of the genes in these clusters for motifs. We used the methods for operon prediction, selecting upstream regions, and applying AlignACE to prokaryotic genomes as described in McGuire et al. [77]. Briefly, because of the presence of operons in prokaryotes, we must choose the upstream region of the operon head rather than the region immediately upstream of the gene of interest. Since it is more important to include the correct region than to erroneously include extra incorrect regions, we use a loose operon definition and include sequences for several different possibilities if there is any ambiguity. We look upstream of our gene of interest and select all intergenic sequences until we encounter either a divergent intergenic region or an intergenic region longer than 300 bp.

Motifs of interest were selected by applying a set of filters: specificity score [77], quality of alignment (AlignACE MAP score) [97], palindromicity [77], and conservation. To determine the degree of conservation, a search matrix was constructed for each motif. Each of the other genomes was searched with this search matrix using CompareACE, and N-way conserved sites were identified. N-way conserved hits are hits identified upstream of orthologous genes in N genomes, where orthology is defined by membership in the same SYNERGY orthogroup. To select interesting motifs we required specificity score < 1e-10, palindromicity > 0.7, MAP score > 5, and at least 8 sites conserved in 8 genomes.

Motifs were compared to a library of search matrices for 9 known *Mtb *motifs (Acr, Crp, CsoR, DosR, FurA, IdeR, KstR, MprAB, and ZurB), as well as a library of 55 *E. coli *motifs [98] and 22 *Corynebacterial *motifs [99]. Comparison of motifs was done using CompareACE [76].

### Defining groups based on expression under different lipids

We separated the experiments in our compendium of *Mtb H37Rv *microarray experiments into separate conditions based on what nutrients were present in their growth conditions (focusing on different lipid conditions, because of the observed importance of lipid metabolism in these organisms). The following categories were used (the number of experiments in each category is shown in parentheses): Palmitic acid (168), Oleic acid (102), Arachidonic and Eicosatetraynoic acids (76), Linoleic acid (41), Eicosatetraynoic acid (13), Ceramide (4), Nordihydroguaiaretic (3), Cholesterol (2), Glucose (1), KstR knockout (1), KstR knockout with cholesterol added (1).

Within each experiment, we extracted a list of genes upregulated 1.5 and 2 standard deviations above the mean. For each category, we considered a gene to be upregulated if it was upregulated in more than 50% of the experiments making up that category. We then searched for genes that were only upregulated under certain conditions or sets of conditions.

We looked at the evolution of these sets of *Mtb H37Rv *genes by taking the other members of their orthogroups across all 31 other organisms. Evolution of these groups can be visualized in our supplementary information http://www.broadinstitute.org/ftp/pub/seq/msc/pub/SYNERGY/index.html.

### d_N_/d_S _Analysis

We used PAML to calculate d_N_/d_S _values according to several different evolutionary models [100,101]. Since orthogroups contain paralogs as well as orthologs, we used the gene trees output from SYNERGY when running PAML. Some orthogroups may contain single-copy orthologs in only two closely related organisms, whereas others could contain paralogs in all 31 organisms.

For the basic model, we used the following parameters: model = 0 and getSE = 1 (to calculate standard errors). This simple evolutionary model gives one value of d_N_/d_S _for each orthogroup, averaged over all lineages as well as all positions in the gene [102]. While this model does not reflect the evolutionary history that has taken place, it is nevertheless a very blunt yet efficient tool for observing selection.

To gain insight into the evolution of the three major clades of the phylogenetic tree we also used a "branch model" where a different d_N_/d_S _value is allowed on a "foreground" branch (but d_N_/d_S _is averaged along positions in the protein) [103,104]. This was done in PAML by using "Model = 2". We compared this model to the basic model using a log-likelihood χ^2 ^test with d.o.f. = 1. For each of the three foreground branches, we used a Bonferroni correction equal to the number of orthogroups present at the branch. We ran this separately for three different "foreground" branches on the phylogenetic tree (labeled in Figure 1): A) The branch leading to the *Mtb *complex; B) The branch leading to pathogenic Mycobacteria; and C) The branch leading to soil-dwelling, non-pathogenic Mycobacteria. The log-likelihood model that we use here compares this branch model to the simple model with a single value of d_N_/d_S _described above, and tests whether the model allowing d_N_/d_S _to differ on the foreground branch fits the data better than the basic model.

Branch-site models allow d_N_/d_S _to vary across branches of the tree and among sites in the protein. We also used the branch-site model of Zhang and Nielsen [100] using Model = 2, NSsites = 2, and fix_blength = 2. We used the model = 0 calculations to determine branch lengths for the branch-site model calculations to save computational time. We compared the results for a subset of the orthogroups with and without fixed tree lengths and determined there was little difference in the results). We chose the same three sets of branches (A-C) that we used for the branch model described above. We compared this model to the corresponding null model using a log-likelihood χ^2 ^test with d.o.f. = 1 [100]. For each of the three foreground branches, we used a Bonferroni correction equal to the number of orthogroups present at the branch. The branch-site model was the most informative.

We calculated the functional group over-representations separately for each functional group dataset. These datasets included 21 COG categories, 168 KEGG categories, 749 metabolic pathways, and 7 additional Mycobacteria-specific groupings (PE genes, PPE genes, toxin-antitoxin genes, DosR regulon, esx genes, *Rv0474 *regulon, and the KstR regulon). We multiplied the hypergeometric p-values by a Bonferroni correction equal to the number of categories or tests performed.

### Generating multi-genome alignments

We constructed whole-genome alignments of all 31 organisms, as well as subsets including only Mycobacterial organisms, and organisms within the *Mtb *complex. These alignments can be downloaded from our website. Our whole genome multiple alignments reveal unannotated stretches of conservation in noncoding regions including transcription factor binding sites in promoter regions, noncoding RNAs, and mis-annotated proteins.

To generate whole-genome multiple alignments, we first aligned the reference genome to each target genome in a pairwise manner. The process of pairwise whole-genome alignment consists of using PatternHunter [105] to identify anchors of local alignment, grouping collinear anchors separated by a limited distance into chains, filtering out smaller chains that shadow larger ones, and finally using LAGAN [106] to globally align the entire chain. Once all genomes have been aligned to the reference, we then identified intervals of the reference that map tightly to a single interval of some or all of the target genomes, and we consider these the endpoints of blocks of multiple alignment. These blocks are generally smaller than any precursor pairwise alignment, because a rearrangement or loss of detectable similarity in any genome will truncate the block for all member genomes. We then ran the multiple aligner MLAGAN on each block. Finally, to facilitate searches for constrained regions of the reference, we projected the blocks onto the reference genome, effectively unwinding all genome rearrangements in the target genomes relative to the reference. We visualized the alignments in the GenomeView browser [64].

### Selecting conserved regions within the alignments

We used Gumby [65] to select conserved regions in our multiple alignments using a value of p < 0.5. In a multiple alignment of all 19 Mycobacterial genomes, we identified 4697 regions of conservation overlapping coding genes in the reference annotation, and 394 regions in intergenic regions.

We also used the method of Ruzzo and Tompa [107] to identify conserved regions. Scores were normalized to the background inferred from the 3 rd-base frequencies. For all *H37Rv *coding sequences, all bases in the third position were extracted from the 31-way multiple alignment. These were concatenated in a new multiple alignment only containing third bases. From this new multiple alignment we calculated the baseline conservation which is used to normalize the conservation scores for the regular alignment. Both sets of highly conserved regions can be viewed as alignment tracks for the GenomeView browser [64], downloadable on our website.

### Predicting RNAs

We predicted regions likely to form RNAs within the conserved intergenic regions of our multiple alignment of 19 Mycobacteria, using Evofold [66]. We divided the intergenic region into 240-bp segments, tiled by 80 bp, to run Evofold. Looking within intergenic regions, we identified 536 regions with Evofold (regions greater than 5 bp in length with length-normalized folding potential score > 0.2).

We examined these 536 regions, as well as the 394 conserved intergenic regions found by Gumby, to see if any of these showed significant expression in our log-phase *Mtb *RNA-seq data. We calculated RPKM [108] values for each of these regions. We examined the regions with RPKM value ≥200 and a number of RNA-seq reads ≥ 20. We eliminated an additional 35 regions which corresponded to known RNAs from the *Mtb *annotation, or RNAs similar to those found in *M. bovis *and *Streptococcus *[60-63], including 26 tRNAs, 2 riboswitches, and 3 found in other organisms.

To select intergenic regions with high levels of expression that do not correspond to UTRs, we also calculated RPKM values for the 100 bp regions of the flanking genes closest to the intergenic regions. We selected those intergenic regions with the highest ratio of the RPKM value of the region of interest (within the intergenic region) to the RPKM of the start/stop of the flanking genes. We also looked for regions with a gap in expression between the gene and the region of interest. This will eliminate many regions that merely correspond to UTRs, and select for regions that are disproportionately expressed within the intergenic region only. We found this method to be most useful for selecting regions of interest, and successfully enriched our top hits for previously known small RNAs. The top 50 predicted RNAs can be viewed as a track in the GenomeView browser (see Supplementary Information).

We further examined log-phase RNA-seq data from *M. smegmatis *to confirm that many of the orthologous regions also show expression in *M. smegmatis*.

### Strain, media, and culture conditions for RNA-seq

*Mycobacterium tuberculosis *H37Rv and *M. smegmatis *were grown at 37°c in 7H9 media supplemented with 10% ADC (Becton Dickinson), 0.2% glycerol and 0.05% Tween 80. For log phase, cells were grown to OD_540 _0.2. Roller bottles were used for culturing *M. tuberculosis*, and shaker flasks for *M. smegmatis*.

### RNA isolation from in vitro cultures for RNA-seq

Bacterial pellet from log-phase cultures of *M. tuberculosis *and *M. smegmatis *were resuspended in TRIzol reagent (Invitrogen) and immediately transferred to 2 ml screw-cap tubes containing 0.1 mm zirconia/silica beads (BioSpec Products). *M. tuberculosis *cells were lysed using a FastPrep-24 bead-beater (MP Biomedicals) 3 times for 30 seconds each at speed 6. *M. smegmatis *cells were lysed using MagNalyser (Roche). Samples were kept on ice for 1 min between pulses. The TRIzol extracted RNA was treated twice with DNAse and further purified using RNAeasy kit (Qiagen).

### Directional mRNA-seq libraries for RNA-seq

We generated mRNA-seq libraries for sequencing on Illumina's GA Sequencer (San Diego, CA). 2 μg purified RNA was depleted of ribosomal RNA using Ambion's MICROBExpress Kit (Austin, TX) as per manufacture's recommended protocol. The enriched mRNA was used to prepare libraries using Illumina's Directional mRNA-seq Library Prep v1.0 protocol. Briefly, 100 ng mRNA was fragmented with cations and heat, end-repaired, adapted by sequential ligation of unique 5-prime and 3-prime adapters, reverse transcribed, PCR amplified, and purified using Agencourt's AMPure Beads (Beverly, MA). The libraries were visualized on an Agilent 2100 Bioanalyzer (Santa Clara, CA) and found to have the expected average fragment length of ~250 bp.

### RNA isolation and Northern Blotting

Total RNA was isolated from Mtb as described previously [109] with minor modifications. Briefly, log-phase cells were pelleted, resuspended in TRIzol (Invitrogen), and transferred to Lysing Matrix B tube (QBiogene). The cells were lysed using MagNalyser (Roche), and RNA extracted with Trizol reagent as instructed by the manufacturer. RNA was treated with Turbo DNase (Ambion) for 30 minutes at 37°C twice and purified further using TRIzol solution and 100% Ethanol.

Total RNA was separated on 10% TBE-Urea acrylamide gels (Bio-Rad) and electroblotted onto Hybond N + membranes (GE Healthcare). After UV cross-linking the membranes were pre-hybridized and hybridized with labeled probes at 48°C as per the DIG manual (Roche). Probe sequences are CGATGGTCGAAAAGGAACTCGATACGGCTATGCGGTTCT (RNA1), AGTTCACGAAACGAAGAAAGAAGCTAAGAAGACATAGGTT (RNA2), GACTGCCAGCAGGCGCCGCGCAATGCGCTTGCAGGACTTC (RNA3), and GGGTGACATGGCTCAGGGAAGCCCGGGCGGGCTGGGACGT (RNA9). After hybridization the membranes were washed twice using a low stringency buffer (2× SSC, 0.1% SDS), and a high stringency buffer (0.1× SSC, 0.1% SDS), for 15 and 5 minutes at 48°C, respectively. The membranes were processed with DIG detection system (Roche) and exposed to X-ray film.

## Abbreviations

Mtb: Mycobacterium tuberculosis; PAH: Polycyclic aromatic hydrocarbons; GO: Gene Ontology; MMR: Mismatch repair.

## Competing interests

The authors declare that they have no competing interests.

## Authors' contributions

AMM performed the analysis and drafted and finalized the manuscript. BW and RR were involved in many aspects of the comparative analysis. STP and SR performed the experimental validation. IW and AR performed the SYNERGY analysis. GD, GKS, RTY, MIM, MJK, and A. Maer provided the *M. tuberculosis *RNA-seq data. TA provided the GenomeView browser. JZ performed the Eficaz and metabolic pathway analyses. JW, PS, and MK constructed multiple alignments. CS worked on the web page. MP worked on motif discovery and network reconstruction. JEG initiated and supervised the study, and revised the manuscript. All authors read and approved the final manuscript.

## Supplementary Material

Additional file 1 **Supplementary Results **[110-130]. Click here for file

Additional file 2 **Additional related tetR family regulators (2-3 copies in each environmental Mycobacterium)**. Click here for file

Additional file 3 **Northern Blots for small RNAs in *M. tuberculosis***. Click here for file

Additional file 4 **Genomes contained in orthogroups**. Click here for file

Additional file 5 **Phylogenetic tree showing bootstrap results**. Click here for file
