# Supplementary material for: Comparative analysis of mycobacterium and related actinomycetes yields insight into the evolution of mycobacterium tuberculosis pathogenesis
Source: BMC Genomics. 2012 Mar 28;13:120. doi: 10.1186/1471-2164-13-120 (PMC3388012; doi:10.1186/1471-2164-13-120)
Supplement: Additional file 1 — Supplementary Results [110-130]. [file 1471-2164-13-120-S1.DOC]

**Supplementary Results**

**d_N_/d_S_**

Complete results for all three models are available in our supplementary information website at <http://www.broadinstitute.org/ftp/pub/seq/msc/pub/SYNERGY/TB_dnds/>. For model=0, we observe that the d_N_/d_S_ values averaged over the entire coding region and the entire phylogenetic tree are rarely above 1, as this signifies sustained evolutionary pressure over long time periods. Of the 14,068 orthogroups containing greater than one member, 12,699 yielded d_N_/d_S_ calculations with reasonably low standard error for the value of d_N_/d_S_ (cutoff requiring SE < d_N_/d_S_). Of these 12,699 orthogroups, the average d_N_/d_S_ value, averaged over the entire tree, was 0.07, which is consistent with previously determined tree averages for bacterial species [110,111]. d_N_/d_S_ values for 3,514 orthogroups with a member in *Mtb* *H37Rv* are listed on our Supplementary Information website. Most of the orthogroups with the highest values of d_N_/d_S_ are PE or PPE family genes. This is expected given the role of PE/PPE genes in antigenic variability and rapid immunoselective pressure from the host [112], and consistent with previous observations [113].

Previous pairwise analyses have showed elevated d_N_/d_S_ values in pathogens [111,114]. We use branch and branch-site models to examine d_N_/d_S_ values on the branches leading to the pathogenic *Mycobacteria*, the branch leading to the *Mtb* group, and the branch leading to the soil dwelling *Mycobacteria*. We observe many genes with selection on each of these three branches (see Supplementary Tables 1 and 2).

For the branch model, a summary of results can be found in Supplementary Table 1, and a listing of orthogroups showing significant difference (either higher or lower d_N_/d_S_) on the foreground branch can be found in the Supplementary Information website. In that values of d_N_/d_S_ are averaged along the length of the protein, this model does not give much power for detecting selection acting on only a small part of the protein. For example, genes containing single point mutations that confer such properties as drug resistance will not be discriminated by this method.

The branch-site model was more useful than the branch model in identifying orthogroups showing selection (see summary in Supplementary Table 2). A greater number of orthogroups were identified as undergoing positive selection on the foreground branches, especially for the branches leading to the pathogenic and soil-dwelling bacteria, than were observed in the simpler branch model. A total of 8% showed significant selection on the branches leading to the pathogenic and soil-dwelling *Mycobacteria*. Interestingly, only 1.6% showed selection on the branch leading to the *Mtb* complex. A listing of orthogroups showing selection can be found in the Supplementary Information website and in Supplementary Tables 2 and 3. Significant over-representations are found for the branches leading to the pathogenic and soil-dwelling *Mycobacteria*.

**Protein evolution: other observations**

*Degradative capabilities of soil-dwelling Mycobacteria*

As expected we see an array of degradation-related categories expanded in the soil-dwelling *Mycobacteria* but not in the pathogenic *Mycobacteria*, reflecting the wide-ranging catabolic capabilities of the soil-dwellers. For example, *M. sp. KMS*, *M. sp. MCS*, *M. vanbaalenii*, and *M. gilvum* are known to degrade polycyclic aromatic hydrocarbons (PAH), and R. jostii RHA1 can degrade polychlorinated biphenyls (PCBs). As expected, we see expansions of many dehydratases, hydrolases, oxidases, oxidoreductases, hydratases, dehydrogenases, and other metabolic enzymes that could contribute to these unusual metabolic capabilities in the soil dwelling *Mycobacteria*. Some particular metabolic pathways, from among the most non-uniform pathways, that are expanded in the soil dwellers include “catechol degradation”, “L-idonate degradation”, “biphenyl degradation”, and “phenolic compound degradation”.

We also see evidence for increased positive selection in genes in the COG category “energy production and conversion”, the KEGG categories “energy metabolism” and “citrate cycle”, and metabolic pathways “energy metabolism” and “TCA variants.” In our branch-site d_N_/d_S_ results, the set of genes showing significant selection is enriched for genes in these categories. This positive selection could be related to the expanded sources of energy in these organisms.

*Evolution of protein families known to be related to pathogenicity*

Toxin-antitoxin genes. Several domains related to toxin-antitoxin genes are among the groups most expanded in the *Mtb* clade: the PFAM PIN domain (PF01850), which is found in the toxic component of toxin-antitoxin genes specific to *Mtbp* [115]; PF02604 (Phd­­_YefM), which complexes with another domain associated with the toxic component; PF02452 (PemK-like protein, found in the toxin component of toxin-antitoxin pairs); PF07704 (*Rv0623*-like transcription factor), which contains a PIN domain and is associated with PSK (post-segregational cell killing) operons [116]; and PF05016 (Plasmid stabilization system protein), which includes toxins related to RelE/ParE [116]. At the root node of the *Mtb* complex (node 33), we see a significant appearance of PIN domains. This is in agreement with previous studies on toxin-antitoxin genes in the *Mtb* complex [115], reporting large numbers of these stress-response elements found only in the *Mtb* complex, likely obtained by horizontal gene transfer. These genes are likely to be involved in responses to stresses encountered in vivo, during hypoxia and phagocytosis by the macrophage.

Esx genes. Several PFAM categories related to *esx* genes are among those most expanded in the *Mtb* clade relative to the nonpathogenic *Mycobacteria*. The PFAM group PF06013 (Proteins of 100 residues with WXG) is expanded. This includes several Esat-6 like proteins, which are known to be involved in virulence and protective immunity in *Mtb* [117], including EsxW, EsxJ, EsxK, EsxP, and EsxA. In addition, the expanded PFAM group PF06359 (Protein of unknown function DUF1066) contains another 5 Esat-6-like proteins (EsxI, EsxL, EsxV, EsxN, and EsxO).

PE / PPE genes. The PFAM PE and PPE families are believed to be surface-associated cell wall proteins involved in providing a diverse antigenic profile and affecting immunity [112]. Within the *Mycobacterial* clade, we see a lot of rearrangements (gains and losses at individual nodes) among the PE and PPE domains. We see significant appearances or duplications at a number of nodes. We see appearances of both PE and/or PPE-containing orthogroups at node 33, the root node of the *Mtb* clade, as well as nodes 36, 38, 39, 42, and 43. We see losses at nodes 17, 24, 27, 28, 34, and 37. This large amount of rearrangement makes sense, given the role of these proteins in maintaining a diverse antigenic profile. However, some of this rearrangement is simply a consequence of the large amount of variability in these genes, which makes it difficult for SYNERGY to properly assort the PE and PPE genes into correct orthogroups. Pittius et al. published a detailed analysis of the evolution of PE and PPE genes and how their expansion is linked to duplications of *esx* clusters [118].

When looking at d_N_/d_S_ values averaged over the entire length of the protein for the entire tree (the simplest model, “Model=0”), we see that most of the proteins with the highest d_N_/d_S_ values are either PE or PPE genes (see Supplementary Information). Given their role in modulating antigenic responses in the host, we would expect that these proteins would show high rates of evolution. However, the difficulties in subdividing these variable groups into correct orthogroups may be the cause of some of these elevated d_N_/d_S_ values.

In our analysis, the PE and PPE gene families were highly enriched for the high- d_N_/d_S_ orthogroups in the basic model, but we were not able to observe selection in the other models because these are found predominantly in the TB clade (hence they are only found on the branch of interest and it is impossible to apply the other two models).

MCE genes. The four *mce* (Mammalian Cell Entry) operons have an important role in host cell invasion, allowing the bacteria to be taken up and survive within the host macrophage [119]. These encode ABC-family transporters, several of which are known to transport fatty acids or cholesterol. We observe that these are expanded in *Mycobacteria*. At the root node of the *Mycobacteria* (node 46), and another nearby node (node 44) we see significant appearances of genes with PFAM *mce* category.

We also see two PFAM categories related to the *mce* genes among the PFAM categories significantly expanded in the *Mycobacteria* relative to the non-*Mycobacteria*: PF02405 (Domain of unknown function DUF140), and PF02470 (*mce* related protein). PF02405 contains four pairs of yrbE proteins in *Mtb* *H37rv* (YrbE1A, YrbE1B, YrbE2A, YrbE2B, YrbE3A, YrbE3B, YrbE4A, and YrbE4B), while PF02470 contains 24 *mce* genes. Each of the four *mce* operons contains two *yrbE* genes and six *mce* genes [120]. The *yrbE* genes have homology to ABC transporter permeases.

In addition to the expansions in this family, we also see evidence for positive selection. In the branch-site evolutionary model, we see six *mce* genes showing significant positive selection on the branch leading to the pathogenic *Mycobacteria*: *mce1D*, *mce1C*, *mce4F*, *mce1A*, *mce4E*, and *mce4D*.

Genes involved in the synthesis of mycolic acid coat. The PFAM group for cyclopropane-fatty-acyl-phospholipid synthase (PF02353) is among the most non-uniform PFAM categories overall, expanded in pathogenic *Mycobacteria*. These genes are involved in producing the mycolic acid coat which is required for successfully evading the host immune system. Evolution of lipid biosynthesis proteins is discussed above.

Other PFAM families expanded in the TB group (compared to the soil dwellers) are also involved in formation of the cell coating. DUF1396 contains a family of lipoproteins expanded in *Mtb* (*LppX, LprF, LprA, and LprG*). One of these, *Rv2945c*, is involved in the synthesis of cell wall components and is known to be involved in the translocation of complex lipids phthiocerol dimycocerosates (DIMs), which are important for virulence [121]. DIMs are thought to protect *Mtb* from reactive nitrogen intermediates produced by macrophages [122,123]. *LprF* is another lipoprotein believed to be involved in signaling of potassium-dependent osmotic stress [124]. *LprA* and *LprG* are TLR2 ligands involved in interacting with the macrophage [125].

Antibiotic resistance genes. The PFAM category PF08077 (“Chloramphenicol resistance gene leader peptide”) is among the PFAM categories most expanded in the other pathogenic *Mycobacteria*, compared to the nonpathogenic *Mycobacteria*. The PFAM group for pentapeptide repeats (PF01469) is also highly expanded in pathogenic *Mycobacteria*. Pentapeptide proteins are composed of tandemly repeated amino acid sequences with a consensus sequence of [S,T,A,V][D,N][L,F][S,T,R][G] [126]. The first known member of this group in *Mtb* (MfpA) is involved in fluoroquinolone resistance by inhibiting a DNA gyrase. The pentapeptide repeat is believed to mimic the structure of DNA.

*Evolution of other transcriptional regulator genes*

There has been the expected amount of loss of regulatory genes in the pathogenic Mycobacteria relative to the larger soil-dwelling Mycobacteria. The number of transcriptional regulators in the genomes across our dataset generally follows the expected power law with n=2, meaning that an organism with twice as many genes would have four times as many regulators [127-130].

Despite the trends towards loss of regulators in the pathogenic Mycobacteria, there are several regulation-related categories that are expanded or only found in the pathogenic Mycobacteria (**Table 2**). These include transcription factors related to *Rv0623*, which are known to be involved with toxin-antitoxin pairs and have a role in pathogenesis [115], and proteins containing a *spoV/abrB*-like domain.

**Supplementary Tables**

**Table S1.** Summary of d_N_/d_S_ results for the Branch Model and Branch-Site Model

|  | | | Branch model | | Branch-site model | |
| --- | --- | --- | --- | --- | --- | --- |
| Branch of interest^a^ | Branch length^b^ | # orthogroups tested^c^ | # significant orthogroups^d^ | % significant | # significant orthogroups ^d^ | % significant |
| 1. Branch leading to *Mtb* complex | 0.40 | 3299 | 77 | 2.3% | 54 | 1.6% |
| 1. Leading to pathogens | 0.47 | 4429 | 137 | 3.1% | 353 | 8.0% |
| 1. Leading to soil-dwellers | 0.32 | 5018 | 106 | 2.1% | 402 | 8.0% |

^a^Branches A-C are labeled in Figure 1.

^b^Branch lengths estimated from Basic Model (model=0 PAML calculation).

^c^Total # of orthogroups tested for the Basic Model was 14,068 (the set of all orthogroups representing >1 species), but not all of these orthogroups were present at the individual branches tested.

^d^The significant orthogroups (Bonferroni-corrected p-value < 0.05) are listed in the Supplementary Information.

**Table S2.** Functional group enrichment (Branch-Site d_N_/d_S_ analysis).

| Branch of interest | Functional group type | Description (check these names) | # significant on this branch^a^ | # in func group | overlap | Bonferroni-corrected p-value |
| --- | --- | --- | --- | --- | --- | --- |
| A: Leading to *Mtb* group | COG | Replication, recombination, and repair | 54 | 197 | 9 | 0.023 |
|  | COG | Secondary metabolites: biosynthesis, transport, and catabolism | 54 | 234 | 10 | 0.020 |
|  |  |  |  |  |  |  |
| B: Leading to pathogens | KEGG | Amino acid metabolism | 288 | 296 | 38 | 0.040 |
|  | Metab. pwys | Carbohydrate biosynthesis | 288 | 116 | 21 | 0.045 |
|  | COG | Energy production and conversion | 288 | 210 | 27 | 0.041 |
|  | COG | Lipid metabolism | 288 | 257 | 32 | 0.027 |
|  |  |  |  |  |  |  |
| C: Leading to soil-dwellers | COG | Energy production and conversion | 270 | 210 | 33 | 5.57e-5 |
|  | KEGG | Amino acid metabolism | 270 | 296 | 39 | 0.00430 |
|  | KEGG | Energy metabolism | 270 | 115 | 23 | 0.000257 |
|  | KEGG | Citrate cycle | 270 | 30 | 9 | 0.0175 |
|  | Metab. pwys | TCA variants | 270 | 52 | 13 | 0.020 |
|  | Metab. pwys | GLNSYN PWY | 270 | 8 | 0 | 0.00115 |
|  | Metab. pwys | Energy metabolism | 270 | 156 | 31 | 1.78e-05 |

^a^This shows the number of orthogroups with members in *Mtb H37Rv* only.

**Supplementary Figures**

**Figure S1.** Summary of SYNERGY results: Orthogroups, categorized by number of organisms represented. The peaks at 8 and 20 genomes represent genes conserved only in the *Mtb* complex, or only in the *Mycobacteria*, respectively.

**Figure S2.** Phylogenetic tree showing bootstrap results. All branches had 100% bootstrap consensus, with the exception of the branches marked with a percentage. The region of the tree within the *Mtb* complex is the most uncertain region of the tree, because of the small branch lengths within this region.

**Figure S3.** KstR and related tetR family regulators in the environmental *Mycobacteria*. This phylogenetic tree shows the relationships between tetR-family proteins related to KstR. This tree was constructed from BLAST hits to *M. tuberculosis* *H37Rv* KstR. This shows that additional related regulators in the environmental *Mycobacteria* are much more closely related to KstR than any other proteins in the *M. tuberculosis*.

**Figure S4.** Full-length gels from our Northern blots.
