# Supplementary figures and images for: Comparative analysis of mycobacterium and related actinomycetes yields insight into the evolution of mycobacterium tuberculosis pathogenesis
Source: BMC Genomics. 2012 Mar 28;13:120. doi: 10.1186/1471-2164-13-120 (PMC3388012; doi:10.1186/1471-2164-13-120)

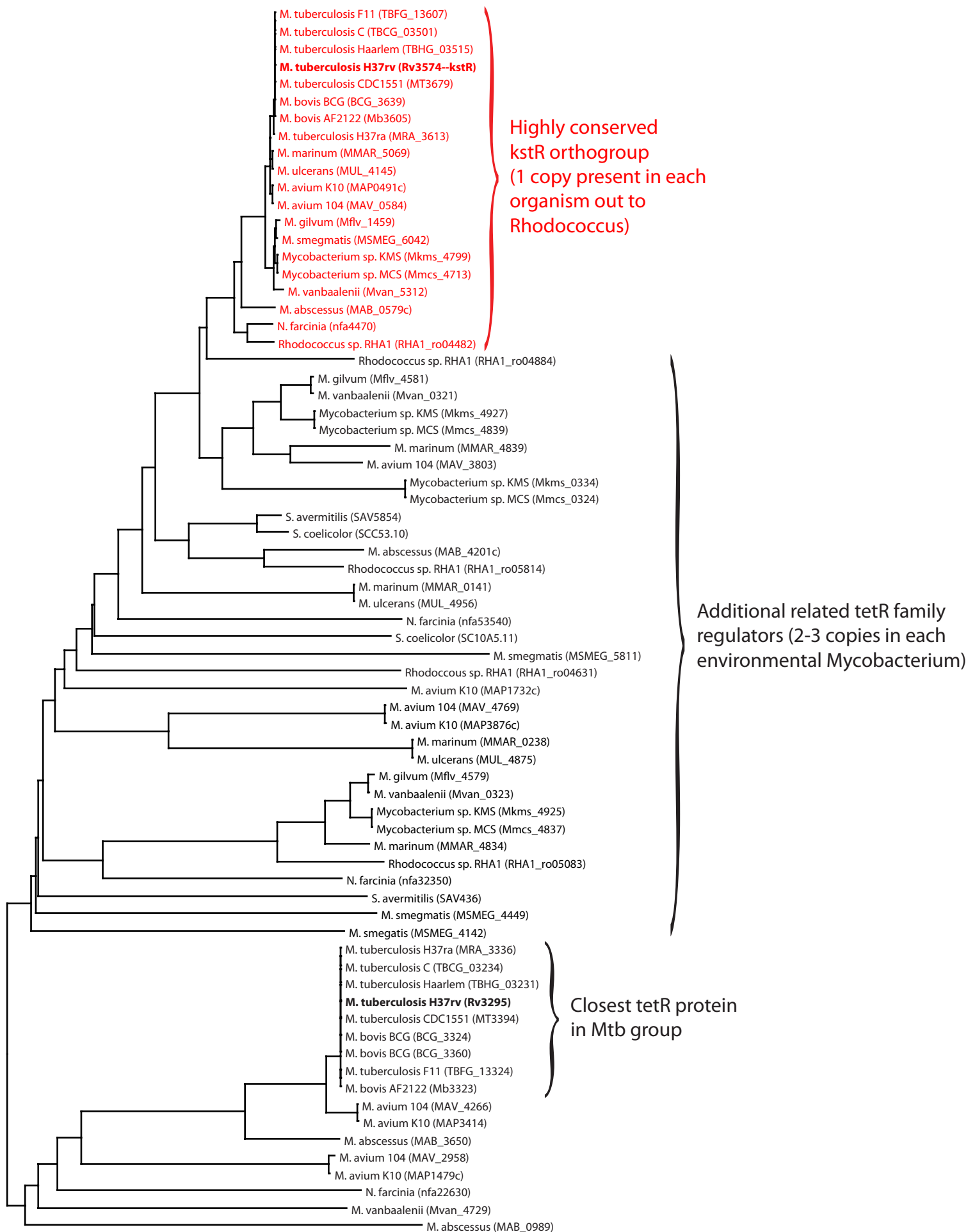

Supplement: Additional file 2 — Additional related tetR family regulators (2-3 copies in each environmental Mycobacterium). [file 1471-2164-13-120-S2.PDF]

# Northern Blots for small RNAs in *M. tuberculosis* : full size

RNA1 (59bp)

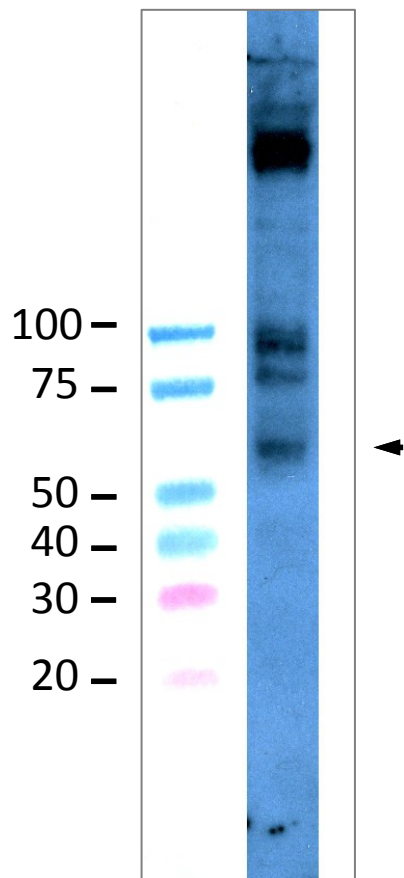

RNA2 (47bp)

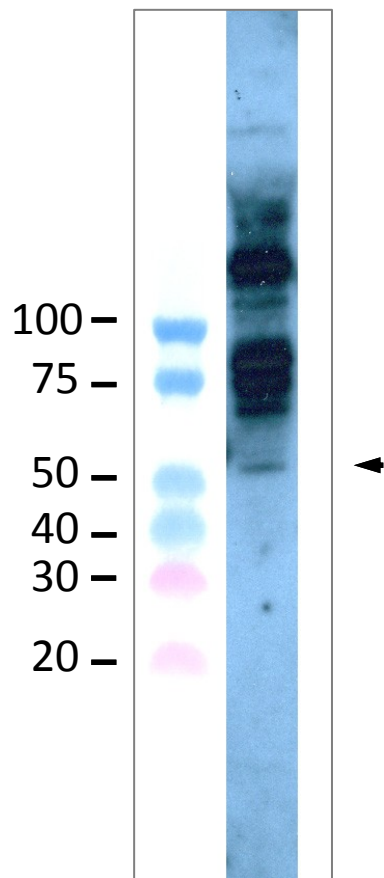

RNA3 (86bp)

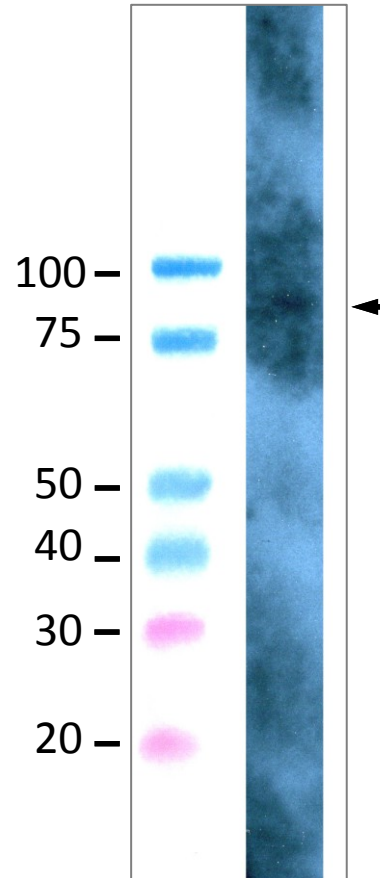

RNA9 (42bp)

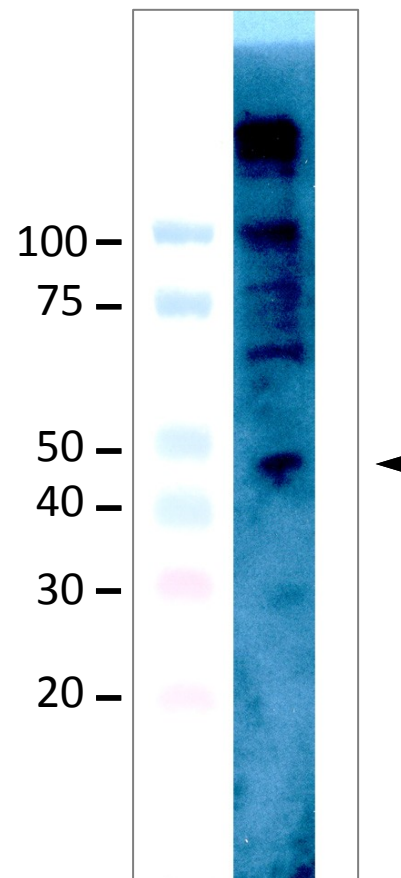

Supplement: Additional file 3 — Northern Blots for small RNAs in M. tuberculosis. [file 1471-2164-13-120-S3.PDF]

# Orthogroups

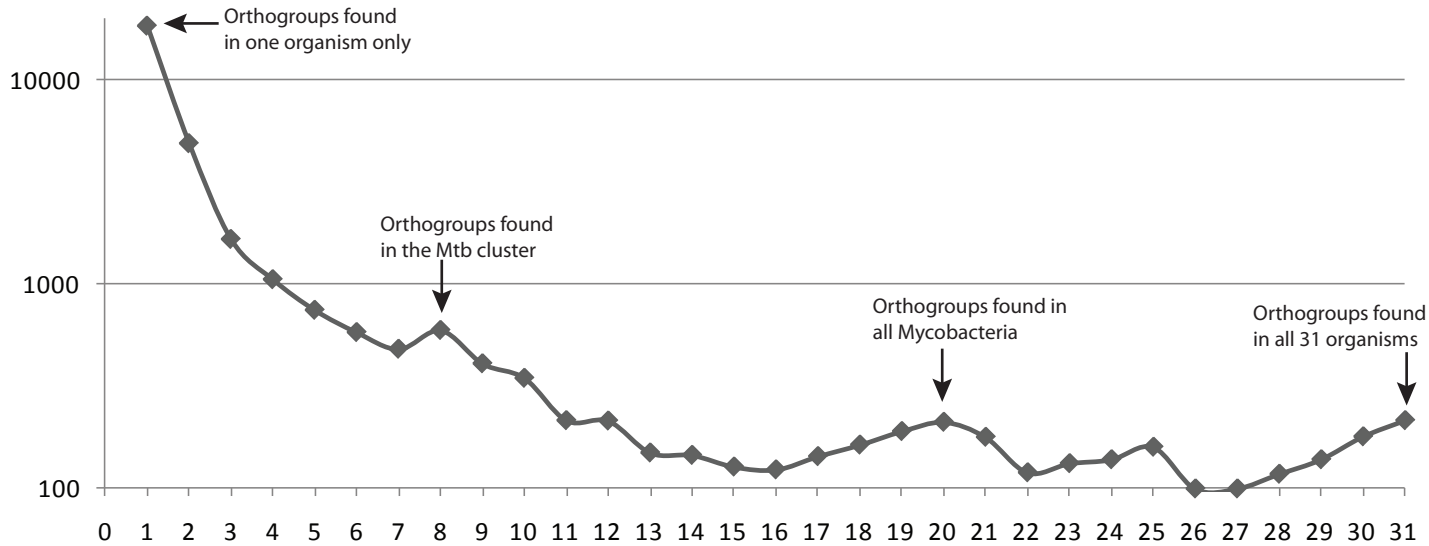

# Genomes contained in orthogroup

Supplement: Additional file 4 — Genomes contained in orthogroups. [file 1471-2164-13-120-S4.PDF]

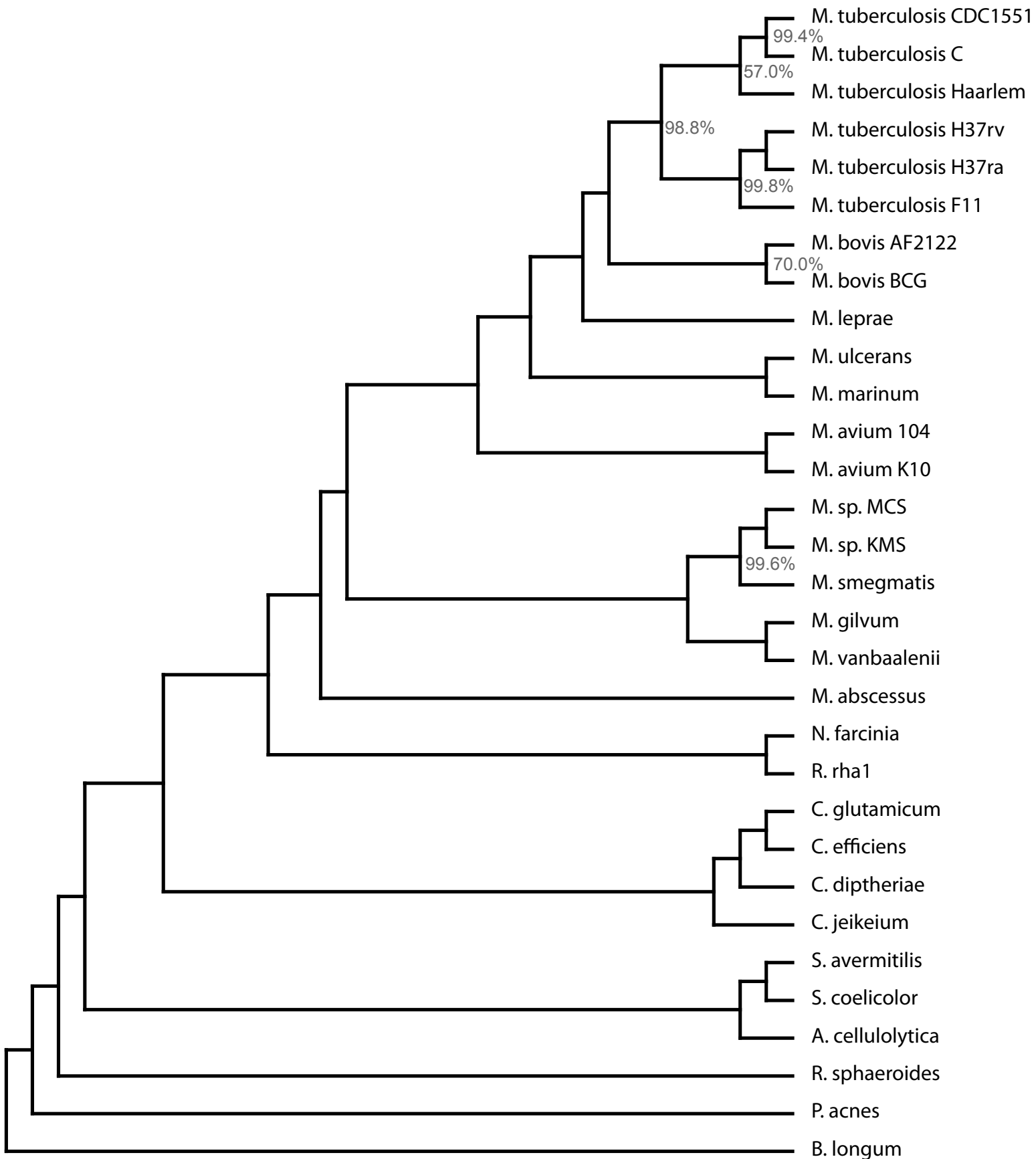

Supplement: Additional file 5 — Phylogenetic tree showing bootstrap results. [file 1471-2164-13-120-S5.PDF]
